# Noniterative Doubles Corrections to the Random Phase and Higher Random Phase Approximations: Singlet and Triplet Excitation Energies

**DOI:** 10.1002/jcc.26074

**Published:** 2019-10-01

**Authors:** Pi A. B. Haase, Rasmus Faber, Patricio F. Provasi, Stephan P. A. Sauer

**Affiliations:** ^1^ Van Swinderen Institute, University of Groningen Nijenborgh 4, 9747AG Groningen The Netherlands; ^2^ Department of Chemistry Technical University of Denmark, Kemitorvet, Bygning 207, 2800 Kgs. Lyngby Denmark; ^3^ Department of Physics, IMIT Northeastern University, Conicet, Corrientes W3404 AAS Argentina; ^4^ Department of Chemistry University of Copenhagen, Universitetsparken 5, 2100 Copenhagen Ø Denmark

**Keywords:** RPA(D), HRPA(D), singlet, triplet, excited states

## Abstract

The second‐order noniterative doubles‐corrected random phase approximation (RPA) method has been extended to triplet excitation energies and the doubles‐corrected higher RPA method as well as a shifted version for calculating singlet and triplet excitation energies are presented here for the first time. A benchmark set consisting of 20 molecules with a total of 117 singlet and 71 triplet excited states has been used to test the performance of the new methods by comparison with previous results obtained with the second‐order polarization propagator approximation (SOPPA) and the third order approximate coupled cluster singles, doubles and triples model CC3. In general, the second‐order doubles corrections to RPA and HRPA significantly reduce both the mean deviation as well as the standard deviation of the errors compared to the CC3 results. The accuracy of the new methods approaches the accuracy of the SOPPA method while using only 10–60% of the calculation time. © 2019 The Authors. *Journal of Computational Chemistry* published by Wiley Periodicals, Inc.

## Graphical abstract

The authors present singlet and triplet excitation energies using existing, doubles‐corrected random phase approximation (RPA(D)), and two new doubles‐corrected higher RPA approximations to the second‐order polarization propagator approximation (SOPPA). The (D)‐methods treat the double excitation manifold of the polarization propagator in a noniterative fashion reducing the computation time compared to SOPPA by 10–60%. The overall performance of the (D)‐methods is comparable to the SOPPA method. Only in cases where the RPA results suffer from triplet instability, the RPA(D) method gives unreliable results.

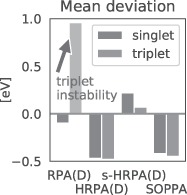

## Introduction

A powerful tool for studying excited states in molecules is the polarization propagator also known as the linear response function.^1^, ^2^ The polarization propagator describes how a system responds to a time‐dependent perturbation and is used for calculating both excitation energies and various response properties. Excitation energies are obtained as the poles of the polarization propagator and the transition moments are the corresponding residues. In practice, the excitation energies can be determined as the eigenvalues of a corresponding eigenvalue problem.^2^, ^3^

The polarization propagator forms the basis of widely used methods for the calculation of excitation energies of various systems and accuracies. Naturally, as the size of a system increases, the accuracy of feasible theoretical methods decreases. This study focuses on methods that give reliable results while still being feasible for chemical interesting systems.

The by far most used method for studying excited states is the time‐dependent density functional theory (TDDFT) method,^4^, ^5^, ^6^ which can handle rather large systems within an acceptable balance between cost and accuracy. However, TDDFT is known to have some difficulties, for example, in describing Rydberg and charge‐transfer states,^6^ handling states with significant double excitation character, and can suffer from triplet instabilities.^7^ If higher accuracy is needed, one often turns to the linear response coupled cluster (CC)‐based methods or related equation‐of‐motion CC methods, which benefit from the well‐defined hierarchy with respect to accuracy that can be defined based on the excitation rank of the operators included in the CC expansion, leading to the coupled cluster singles (CCS), coupled cluster singles and doubles (CCSD), coupled cluster singles, doubles and triples (CCSDT) models, and so forth.^8^, ^9^, ^10^, ^11^, ^12^

An alternative strategy is to define appropriate approximations to the polarization propagator in a consistent manner with the aid of Møller–Plesset perturbation theory by evaluating the propagator with respect to the order in the fluctuation potential to which the propagator, excitation energies, and transition moments are correct. This allows for the construction of a hierarchy with respect to accuracy, similar to that of the CC‐based methods. Two related but not equal approaches exist for this: the algebraic–diagrammatic construction approaches^13^, ^14^ and the *n*th‐order polarization propagator approximations.^2^

In the zeroth‐order polarization propagator approximation, excitation energies are simply equal to orbital energy differences. The first‐order polarization propagator approximation is also called time‐dependent Hartree–Fock or the random phase approximation (RPA).^15^, ^16^ The wave function which is used to construct the RPA equations is the Hartree–Fock self‐consistent field wave function and RPA excitation energies consist of single excitation (and de‐excitation) contributions only. The working equation of RPA is very similar to that of TDDFT and the two methods thus suffer from the same issues, however, to a different extent, as mentioned above.

In the second‐order polarization propagator approximation (SOPPA), the wave function employed is the second‐order Møller–Plesset wave function and both single and double excitation (and de‐excitation) operators are included.^2^, ^17^ Excitation energies dominated by single excitations are in SOPPA correct through second order, whereas pure double excitations are correct only through zeroth order, corresponding to orbital energy differences. Solving the full SOPPA equations becomes demanding for large systems due to the rapidly increasing number of double excitations with increasing number of orbitals. Thus, a number of methods have been developed which aim to capture some of the effects from SOPPA while reducing the size of the problem.

Such approximations start from the RPA problem with the oldest being the higher RPA method (HRPA),^18^, ^19^, ^20^ a precursor to SOPPA. In HRPA, the second‐order contributions, which are included in SOPPA, to the single excitation manifold are added to the RPA equations. The HRPA method is thus somewhere between a first‐order and a second‐order method and the performance is in fact worse than RPA due to the tendency to overestimate excitation energies considerably.^19^, ^21^, ^22^

A more successful approximation is the doubles‐corrected RPA method (RPA(D)), which includes the second‐order contributions from SOPPA in a noniterative fashion on top of a normal RPA calculation using pseudoperturbation theory.^23^ The RPA(D) method is superior to the RPA and HRPA methods,^23^, ^24^, ^25^, ^26^, ^27^ but a drawback is that if the RPA solution suffers from the so‐called singlet and in particular triplet instabilities,^28^ the RPA(D) result will be unreliable which will be demonstrated in this study. We will show that this problem can be circumvented by combining the apparent stability of the HRPA method with the noniterative doubles correction and we call this method the HRPA(D) method. Other examples of circumventing the issue of triplet instabilities in RPA are to employ the well‐known Tamm–Dancoff approximation (TDA),^29^, ^30^ resulting in the configuration interaction singles (CIS) method or to use the recent particle–particle RPA method.^30^

For testing the new methods, we use the popular benchmark set constructed by Thiel et al.,^31^, ^32^, ^33^ which consists of 28 medium‐sized molecules intended to resemble organic chromophores. Because this benchmark set considers only valence excited states, a separate study would be needed to investigate the performance of the new methods for Rydberg excited states. In the original study, singlet and triplet vertical excitation energies were calculated at the CC2, CCSD, CC3, and CASPT2 levels using the medium‐sized basis set TZVP with the conclusion that, compared to the CASPT2 results, the CC3 method was superior to the CC2 and CCSD methods.^31^ Although CC2 performed best for singlet excitation energies,^26^, ^31^ CCSD performed slightly better for triplet excitation energies.^31^ In a recent study by Loos et al.^34^ on 106 different singlet and triplet excited states, the CCSD method was found to perform slightly better than CC2 and again the CC3 method was found to perform very well‐giving results almost identical to the CCSDT and coupled cluster singles, doubles, triples and quadruples (CCSDTQ) methods. In this study, we have chosen to use the CC3 results as reference values.

The performances of the SOPPA‐based methods, RPA(D), SOPPA, and SOPPA(CCSD), where the MP2 amplitudes were replaced by CCSD amplitudes, were recently investigated by Sauer et al.^27^ using the benchmark set of Thiel et al. Singlet excitation energies were calculated with the TZVP and also the larger aug‐cc‐pVTZ basis set. Comparing the SOPPA/aug‐cc‐pVTZ singlet excitation energies to the CC3/aug‐cc‐pVTZ energies showed that the SOPPA results on average underestimated the energies by 0.45 eV. The RPA(D) results showed quite different behavior because they were distributed more around the CC3 results, leading to an average underestimation of 0.16 eV. The consistency of the RPA(D) results was, however, lower than the SOPPA results as indicated by a larger standard deviation.

Triplet excitation energies were calculated using the SOPPA and SOPPA(CCSD) methods and the TZVP basis set with the conclusions very similar to those of the singlet excitation energy results. In this study, we extend that study to include also triplet excitation energies on the RPA(D) level.

Compared to the CC3 results, the performances of the SOPPA‐based methods are worse than the CC2 results presented in Ref. 31. As mentioned in Refs. 27 and 26, the standard deviations of the CC2 and SOPPA results are similar whereas the mean deviation of the CC2 results is smaller than SOPPA. The comparison of the SOPPA methods with TDDFT methods is rather difficult as TDDFT results vary significantly with the choice of exchange correlation functional.^35^ A comparison with the widely used B3LYP functional shows that although SOPPA underestimates the results slightly more, the consistency of the SOPPA results is significantly better.

The benchmark set of Thiel et al., or a subset hereof, has been used by various authors to study vertical excitation energies using semiempirical methods,^36^ various ADC methods,^37^, ^38^, ^39^ the PNO‐CC2 method^40^ as well as a recent implementation of multiconfigurational short‐range DFT.^41^ The applicability of the RPA(D) as well as the new HRPA(D) method to linear response properties has recently been demonstrated by Schnack‐Petersen et al.^42^ in the specific case of NMR indirect nuclear spin–spin coupling constants. In this study, we explore now the reasons for the good performance of RPA(D) and HRPA(D) for coupling constants by investigating the performance of these methods in the calculation of electronic excitation energies.

## Theory

The foundation for the methods introduced in this study is the polarization propagator or linear response function. Considering the spectral representation of the polarization propagator, it can be shown that the frequencies which correspond to the poles of the polarization propagator are the vertical excitation energies, {*ω*_*j*_}, and considering the matrix form of the polarization propagator, it can be shown that *ω*_*j*_ can be obtained by solving the generalized eigenvalue problem^2^, ^3^:

(1) $${\mathrm{ER}}_{j}=\omega_{j}{\mathrm{SR}}_{j}.$$

Atomic units are used throughout this study.

The electronic Hessian and the overlap matrices are given as the matrices:

(2) $$E=〈0|\left[h,\left[\hat{H},\tilde{h}\right]\right]|0〉,$$

(3) $$S=〈0|\left[h,\tilde{h}\right]|0〉,$$

where the Hamiltonian consists of the sum of the Fock operator and the fluctuation potential:

(4) $$\hat{H}=\hat{F}+\hat{V}.$$

Based on the partitioning of the Hamiltonian in eq. (4), the wave function, ∣0〉, (and the **E** and **S** matrices) can be expanded in a perturbation series in V according to Møller–Plesset perturbation theory.^43^ In practice, a truncated version of ∣ 0〉 is used as reference state in the evaluation of the matrix elements of the **E** and **S** matrices.

The column vector, **h**, is a complete operator manifold where **h** ∣ 0〉 =  ∣ **n**〉 should generate all excited states of the system and $\tilde{h}$ is the transpose.^2^ It has been shown that the elements of **h** can be constructed of single, double, and so on, excitation (and de‐excitation) operators^2^, ^44^:

(5) $$h=\left(\begin{array}{c} h_{1}\\ h_{2}\\ \vdots \end{array}\right),$$

where

(6) $$h_{1}=\left(\begin{array}{c} q_{1}^{†}\\ q_{1} \end{array}\right),\;h_{2}=\left(\begin{array}{c} q_{2}^{†}\\ q_{2} \end{array}\right),\ldots ,$$

Here, $q_{1}^{†}$ (**q**_1_) denotes a vector containing all single excitation (single de‐excitation) operators, $\left\{q_{\mathrm{ai}}^{†}\right\}$ ({*q*_*ai*_}), and $q_{2}^{†}$ (**q**_2_) contains all double excitation (double de‐excitation) operators, $\left\{q_{\mathrm{ai}}^{†}q_{\mathrm{bj}}^{†}\right\}$ ({*q*_*ai*_ *q*_*bj*_}), where the indices *i*, *j*, *k*, *l* and *a*, *b*, *c*, *d*, refer to occupied and unoccupied orbitals, respectively.

The operator manifold, **h**, is in principle of infinite dimension and has to be truncated for approximations to the polarization propagator.^2^, ^3^ These approximations are characterized by the order in terms of the fluctuation potential, denoted below with a superscript in brackets, through which the excitation energies of mainly particle–hole character, the transition moments or the polarization propagator itself are evaluated.

As a consequence of the definition of the excitation operator in eq. (5), the eigenvectors of the generalized eigenvalue problem, eq. (1), have the following structure:

(7) $$R_{j}=\left(\begin{array}{c} R_{j}^{\mathrm{ph}}\\ R_{j}^{\mathrm{hp}}\\ R_{j}^{2p2h}\\ R_{j}^{2h2p} \end{array}\right),$$

where ph (hp) denotes the single excitation (de‐excitation) manifold and 2p2h (2h2p) denotes the double excitation (de‐excitation) manifold.

In this study, the following well‐known approximations will be employed and discussed in the sections below: the first‐order polarization propagator, better known as the RPA, the HRPA, and the SOPPA. Furthermore, the noniterative doubles corrections to the RPA and HRPA methods, RPA(D) and HRPA(D), will be introduced. The HRPA(D) method for excitation energies is formulated for the first time in this study.

### Random phase approximation

In RPA, the operator manifold consists of the single (de‐)excitation operators, **h**_1_, and the reference state is the Hartree–Fock state.^2^ The RPA **E** and **S** matrices consist thus of the single excitation block only,

(8) $$E^{\mathrm{RPA}}=\left(\begin{array}{cc} A^{\left(0,1\right)} & B^{\left(1\right)*}\\ B^{\left(1\right)} & A^{\left(0,1\right)*} \end{array}\right)\;\text{and}\;S^{\mathrm{RPA}}=\left(\begin{array}{cc} S^{\left(0\right)} & 0\\ 0 & -S^{\left(0\right)*} \end{array}\right),$$

see eq. (11), (12) and (16) for expressions for the **A**, **B**_,_ and **S** matrices.

### Higher RPA method

In the HRPA method, second‐order contributions to the **A**, **B**_,_ and **S** matrices are included.^19^, ^20^ This is equivalent to letting **h**_1_ again span the excitation operator manifold, employing the second‐order Møller–Plesset wave function as reference state and keeping only terms through second order in the fluctuation potential in the **E** and **S** matrices.

(9) $$E^{\text{HRPA}}=\left(\begin{array}{cc} A^{\left(0,1,2\right)} & B^{\left(1,2\right)*}\\ B^{\left(1,2\right)} & A^{\left(0,1,2\right)*} \end{array}\right)\;\text{and}\;S^{\text{HRPA}}=\left(\begin{array}{cc} S^{\left(0,2\right)} & 0\\ 0 & -S^{\left(0,2\right)*} \end{array}\right).$$

### Second‐order polarization propagator approximation

It has been shown that in order for the polarization propagator to be correct through second order (for particle–hole dominated excitations), the **h**_1_ and **h**_2_ operator manifolds are needed. Furthermore, the reference state must be the second‐order Møller–Plesset wave function.^2^, ^17^ The SOPPA matrices then become

(10) $$E^{\text{SOPPA}}=\left(\begin{array}{cccc} A^{\left(0,1,2\right)} & B^{\left(1,2\right)*} & {\tilde{C}}^{\left(1\right)} & 0\\ B^{\left(1,2\right)} & A^{\left(0,1,2\right)*} & 0 & {\tilde{C}}^{\left(1\right)*}\\ C^{\left(1\right)} & 0 & D^{\left(0\right)} & 0\\ 0 & C^{\left(1\right)*} & 0 & D^{\left(0\right)*} \end{array}\right)\;\text{and}\;\;S^{\text{SOPPA}}=\left(\begin{array}{cccc} S^{\left(0,2\right)} & 0 & 0 & 0\\ 0 & -S^{\left(0,2\right)*} & 0 & 0\\ 0 & 0 & 1 & 0\\ 0 & 0 & 0 & -1 \end{array}\right).$$

The elements of the matrices which constitute the electronic Hessian matrix are defined as

(11) $$A_{\mathrm{ai},\mathrm{bj}}^{\left(0,1,2\right)}={〈0|\left[q_{\mathrm{ai}},\left[\hat{H},q_{\mathrm{bj}}^{†}\right]\right]|0〉}^{\left(0,1,2\right)},$$

(12) $$B_{\mathrm{ai},\mathrm{bj}}^{\left(1,2\right)}={〈0|\left[q_{\mathrm{ai}},\left[\hat{H},q_{\mathrm{bj}}\right]\right]|0〉}^{\left(1,2\right)},$$

(13) $$C_{\text{aibj},\mathrm{ck}}^{\left(1\right)}={〈0|\left[q_{\mathrm{ai}}q_{\mathrm{bj}},\left[\hat{H},q_{\mathrm{ck}}^{†}\right]\right]|0〉}^{\left(1\right)},$$

(14) $${\tilde{C}}_{\mathrm{ck},\text{aibj}}^{\left(1\right)}={〈0|\left[q_{\mathrm{ck}},\left[\hat{H},q_{\mathrm{ai}}^{†}q_{\mathrm{bj}}^{†}\right]\right]|0〉}^{\left(1\right)},$$

(15) $$D_{\text{aibj},\text{ckdl}}^{\left(0\right)}={〈0|\left[q_{\mathrm{ai}}q_{\mathrm{bj}},\left[\hat{H},q_{\mathrm{ck}}^{†}q_{\mathrm{dl}}^{†}\right]\right]|0〉}^{\left(0\right)},$$

and the overlap matrix between single excited states is

(16) $$S_{\mathrm{ai},\mathrm{bj}}^{\left(0,2\right)}={〈0|\left[q_{\mathrm{ai}},q_{\mathrm{bj}}^{†}\right]|0〉}^{\left(0,2\right)}.$$

The explicit expressions for the above matrices depend of course on the explicit form of the excitation operators and can be found in the literature.^2^, ^3^, ^17^, ^45^, ^46^

### Noniterative second‐order doubles corrections

The noniterative second‐order doubles‐corrected methods are based on a pseudoperturbation expansion of the SOPPA **E** and **S** matrices.^23^

(17) $$E=E^{\left\{0\right\}}+E^{\left\{1\right\}}+E^{\left\{2\right\}},$$

(18) $$S=S^{\left\{0\right\}}+S^{\left\{1\right\}}+S^{\left\{2\right\}},$$

where the curly brackets indicate the order in the pseudoperturbation theory.

By expanding the eigenvalue in eq. (1) in the pseudoperturbation, the first‐order correction turns out to be zero and the total excitation energy, which is correct through second order, is then given by^23^

(19) $$\begin{array}{l} \omega_{j}^{\left\{0\right\}}+\omega_{j}^{\left\{2\right\}}=R_{j}^{\left\{0\right\}†}\left(E^{\left\{0\right\}}+E^{\left\{2\right\}}-\omega_{j}^{\left\{0\right\}}S^{\left\{2\right\}}\right)R_{j}^{\left\{0\right\}}\\ \;-R_{j}^{\left\{0\right\}†}\left(E^{\left\{1\right\}}-\omega_{j}^{\left\{0\right\}}S^{\left\{1\right\}}\right){\left(E^{\left\{0\right\}}-\omega_{j}^{\left\{0\right\}}S^{\left\{0\right\}}\right)}^{-1}\left(E^{\left\{1\right\}}-\omega_{j}^{\left\{0\right\}}S^{\left\{1\right\}}\right)R_{j}^{\left\{0\right\}}, \end{array}$$

where $R_{j}^{\left(0\right)}$ is the RPA eigenvector augmented with a double‐excitation part wherein all elements are zero:

(20) $$R_{j}^{\left\{0\right\}}=\left(\begin{array}{c} R_{j}^{\mathrm{ph},\mathrm{RPA}}\\ R_{j}^{\mathrm{hp},\mathrm{RPA}}\\ 0\\ 0 \end{array}\right).$$

Here “ph” denotes the excitation manifold and “hp” denotes the de‐excitation manifold of the RPA eigenvector.

Equation (19) is easily implemented in an existing propagator code which is based on the linear transformation of trial vectors. The difference between RPA(D) and HRPA(D) lies in the definition of the **E**^{0}^, **E**^{1}^, and **E**^{2}^ matrices as will be discussed in the following sections.

### Doubles‐corrected RPA method

In RPA(D), the partitioning of the SOPPA **E** and **S** matrices looks like

(21) $$\begin{array}{l} E^{\left\{0\right\}}=\left(\begin{array}{cccc} A^{\left(0,1\right)} & B^{\left(1\right)*} & 0 & 0\\ B^{\left(1\right)} & A^{\left(0,1\right)*} & 0 & 0\\ 0 & 0 & D^{\left(0\right)} & 0\\ 0 & 0 & 0 & D^{\left(0\right)*} \end{array}\right),\;E^{\left\{1\right\}}=\left(\begin{array}{cccc} 0 & 0 & {\tilde{C}}^{\left(1\right)} & 0\\ 0 & 0 & 0 & {\tilde{C}}^{\left(1\right)*}\\ C^{\left(1\right)} & 0 & 0 & 0\\ 0 & C^{\left(1\right)*} & 0 & 0 \end{array}\right),\;\text{and}\;\\ E^{\left\{2\right\}}=\left(\begin{array}{cccc} A^{\left(2\right)} & B^{\left(2\right)*} & 0 & 0\\ B^{\left(2\right)} & A^{\left(2\right)*} & 0 & 0\\ 0 & 0 & 0 & 0\\ 0 & 0 & 0 & 0 \end{array}\right), \end{array}$$

and

(22) $$S^{\left\{0\right\}}=\left(\begin{array}{cccc} S^{\left(0\right)} & 0 & 0 & 0\\ 0 & -S^{\left(0\right)*} & 0 & 0\\ 0 & 0 & 1 & 0\\ 0 & 0 & 0 & -1 \end{array}\right),\;S^{\left\{1\right\}}=0,\;\text{and}\;S^{\left\{2\right\}}=\left(\begin{array}{cccc} S^{\left(2\right)} & 0 & 0 & 0\\ 0 & -S^{\left(2\right)*} & 0 & 0\\ 0 & 0 & 0 & 0\\ 0 & 0 & 0 & 0 \end{array}\right).$$

The RPA(D) excitation energy thus consists of three types of contributions. A zeroth‐order part which is just the RPA excitation energy:

(23) $$\omega_{j}^{\left\{0\right\}}=R_{j}^{\left\{0\right\}†}E^{\left\{0\right\}}R_{j}^{\left\{0\right\}}=\omega_{j}^{\mathrm{RPA}},$$

a second‐order correction from the particle–hole part:

(24) $$\omega_{j,\mathrm{ph}}^{\left\{2\right\}}=R_{j}^{\left\{0\right\}†}\left(E^{\left\{2\right\}}-\omega_{j}^{\left\{0\right\}}S^{\left\{2\right\}}\right)R_{j}^{\left\{0\right\}},$$

and a second‐order correction from the two‐particle–two‐hole part:

(25) $$\omega_{j,2p2h}^{\left\{2\right\}}=-R_{j}^{\left\{0\right\}†}E^{\left\{1\right\}}{\left(E^{\left\{0\right\}}-\omega_{j}^{\left\{0\right\}}S^{\left\{0\right\}}\right)}^{-1}E^{\left\{1\right\}}R_{j}^{\left\{0\right\}}.$$

Due to the structure of the **E**^{1}^ matrices, only the 2p2h part of the **E**^{0}^ and **S**^{0}^ matrices will contribute to $\omega_{j,2p2h}^{\left\{2\right\}}$ which allows for a simplification of the expression as the **D**^{0}^ and the 2p2h part of the **S**^{0}^ matrices are diagonal:

(26) $$\omega_{j,2p2h}^{\left\{2\right\}}=-\sum_{\mu_{2}}\frac{Y_{j,\mu_{2}}Y_{j,\mu_{2}}}{\omega_{\mu_{2}}-\omega_{j}^{\left\{0\right\}}},$$

where $\omega_{\mu_{2}}$ are the diagonal elements of the **D**^{0}^ matrix and **Y**_*j*_ is the transformed RPA eigenvector:

(27) $$Y_{j}=E^{\left\{1\right\}}R_{j}^{\left\{0\right\}}.$$

### Doubles‐corrected HRPA method

In HRPA(D), the **E**^{2}^ and **S**^{2}^ matrices are zero and the partitioning of the SOPPA **E** and **S** matrices then looks like

(28) $$E^{\left\{0\right\}}=\left(\begin{array}{cccc} A^{\left(0,1,2\right)} & B^{\left(1,2\right)*} & 0 & 0\\ B^{\left(1,2\right)} & A^{\left(0,1,2\right)*} & 0 & 0\\ 0 & 0 & D^{\left(0\right)} & 0\\ 0 & 0 & 0 & D^{\left(0\right)*} \end{array}\right),\;E^{\left\{1\right\}}=\left(\begin{array}{cccc} 0 & 0 & {\tilde{C}}^{\left(1\right)} & 0\\ 0 & 0 & 0 & {\tilde{C}}^{\left(1\right)*}\\ C^{\left(1\right)} & 0 & 0 & 0\\ 0 & C^{\left(1\right)*} & 0 & 0 \end{array}\right),\;\text{and}\;E^{\left\{2\right\}}=0,$$

and

(29) $$S^{\left\{0\right\}}=\left(\begin{array}{cccc} S^{\left(0,2\right)} & 0 & 0 & 0\\ 0 & -S^{\left(0,2\right)*} & 0 & 0\\ 0 & 0 & 1 & 0\\ 0 & 0 & 0 & -1 \end{array}\right),\;S^{\left\{1\right\}}=0,\;\text{and}\;\;S^{\left\{2\right\}}=0.$$

Again, the zeroth‐order part of the HRPA(D) excitation energy is just the HRPA excitation energy

(30) $$\omega_{j}^{\left\{0\right\}}=R_{j}^{\left\{0\right\}†}E^{\left\{0\right\}}R_{j}^{\left\{0\right\}}=\omega_{j}^{\text{HRPA}},$$

while the second‐order correction to the particle–hole part vanishes due to the definition of the **E**^{2}^ and **S**^{2}^ matrices and the second‐order correction to the two‐particle–two‐hole part takes the same form as in the case of RPA(D) [see eq. (25)].

### Shifted HRPA(D)

It will be clear from the results of this benchmark study, that it is advantageous to include a second‐order contribution to the particle–hole part coming from the **S**^{2}^ matrix of RPA(D), eq. (22), similar to RPA(D) in the form of

(31) $$\omega_{j,\mathrm{ph}}^{\left\{2\right\}*}=-\left(\omega_{j}^{\left\{0\right\}}R_{j}^{\left\{0\right\}†}S_{\mathrm{RPA}\left(D\right)}^{\left\{2\right\}}R_{j}^{\left\{0\right\}}\right).$$

The second‐order correction to the particle–hole part is consequently accounted for twice; once in the iterative solution of the HRPA equation and once through this noniterative correction. While there is no theoretical reason why this additional correction should improve the HRPA(D) excitation energies, the results presented in this study show that the systematic underestimation of HRPA(D) energies is eliminated upon inclusion of the correction in eq. (31). As the HRPA(D) energies are shifted by overall ~0.5 eV, we will call this extension of HRPA(D) the shifted HRPA(D) (s‐HRPA(D)) method.

### Atomic orbital integral direct implementation

The new methods, RPA(D) for excited triplet states and HRPA(D) and s‐HRPA(D) for excited singlet and triplet states, have been implemented in the existing atomic orbital‐based integral direct SOPPA code which is part of the DALTON program.^46^, ^47^ The solution of the generalized eigenvalue problem, eq. (1), is obtained by solving a reduced eigenvalue problem for a small set of only the lowest eigenvalues.^45^, ^48^, ^49^ This approach avoids the construction of the full **E** and **S** matrices by implementing directly the result of linear transformations of trial vectors by these matrices.^45^, ^48^, ^49^ The same linear transformation algorithm is easily used to compute the noniterative doubles corrections in RPA(D), HRPA(D), and s‐HRPA(D). The involved expressions are calculated directly from integrals over the basis functions (atomic orbitals) and this formalism is thus referred to as an integral‐direct method, originally formulated by Koch et al.^50^

## Computational Details

All calculations were carried out with a local development version of the Dalton program which includes all new methods presented here.^47^

**Figure 1:**
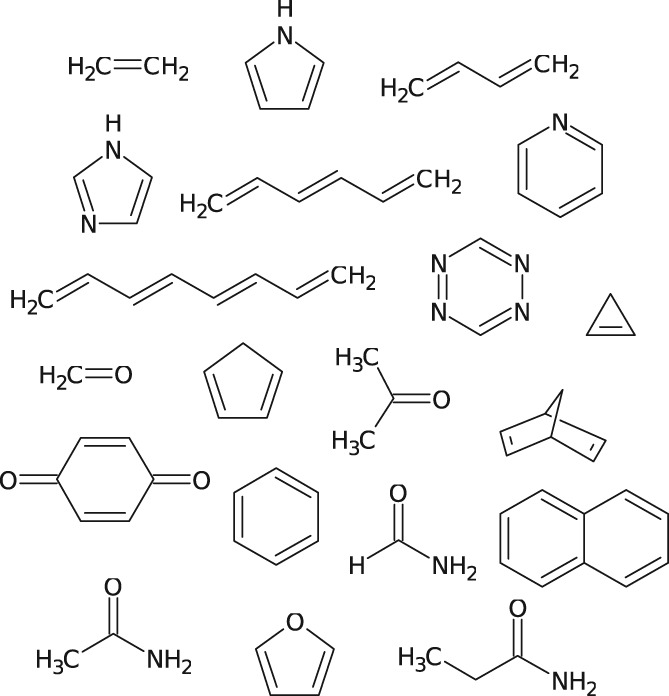
Structures of the 20 molecules used in this study.

The benchmark set used in this study consists of the 20 molecules from the larger set of 28 molecules originally presented in Ref. 31, 32, 33, for which triplet excitation energies were reported at the CC3/TZVP level. The error analysis in this study is made in relation to these CC3 results. The test set is shown in Figure 1 and it consists of aliphatic unsaturated hydrocarbons, aromatic hydrocarbons, and heterocycles as well as a few aldehydes, ketones, and amides. All of the structures had previously been optimized at the MP2/6‐31G* level and can be found in the Supporting Information of Ref. 31.

Detailed information on the studied singlet and triplet excited states can be found in the Supporting Information Tables S2 and S5, respectively. The set of excited states consists of valence excited states of *σ* → π*, *π* → π*, and *n* → π* character. The states have been identified by comparison of the eigenvectors with the eigenvectors of the reference calculations. In RPA and HRPA, the order of the states can deviate from those of SOPPA, as seen, for example, in Ref. 26. The particular cases have been marked with a footnote in Supporting Information Tables S2 and S5.

As the CC3 reference values are available for the TZVP basis set, this medium‐sized basis set^51^, ^52^ was also used in the present study. The number of basis functions with the TZVP basis set ranges from 62 (ethene) to 238 (naphthalene). Results with the TZVP basis were obtained with frozen core orbitals. The effect of freezing the core orbitals has been found in this and previous studies^46^ to be less than 0.01 eV for RPA and SOPPA excitation energies and can thus be considered to be negligible.

## Benchmark Results

### Singlet excited states

In this section, the performance of the two new methods, HRPA(D) and s‐HRPA(D), for determining singlet excitation energies will be compared to the well‐known methods RPA, RPA(D), HRPA, and SOPPA. The RPA and HRPA results for this test set are presented for the first time here, whereas the RPA(D) and SOPPA results have been presented previously^27^ but are included here for clarity. The comparison in terms of computing time will be discussed at the end of this paper.

**Figure 2:**
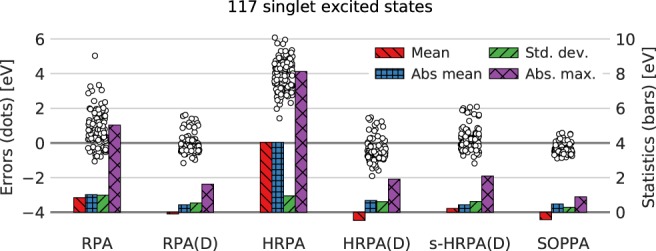
Distributions of errors (circles, left axis) relative to CC3 results (calculated as method—CC3) and statistical measures (bars, right axis) of 117 singlet excited states. RPA(D) and SOPPA results are taken from Ref. 27. Numerical values for statistical measures can be found in Table 1. TZVP basis set, frozen core. [Color figure can be viewed at http://wileyonlinelibrary.com]

In Figure 2, the distribution of errors (left axis) with respect to the CC3 results are shown for the methods mentioned earlier. Furthermore, a statistical analysis of the errors (right axis) is shown as bars in terms of mean deviation, absolute mean deviation, standard deviation, and maximum deviation. The values of these measures are also listed in Table 1.

**Table 1:** Statistical measures (eV) as used in Figure 2 of 117 singlet excited states.

	RPA	RPA(D)	HRPA	HRPA(D)	s‐HRPA(D)	SOPPA
Count	117	117	117	117	117	117
Mean	0.84	−0.1	4.03	−0.47	0.22	−0.42
Absolute mean	1.02	0.43	4.03	0.68	0.43	0.48
Standard deviation	0.99	0.54	0.95	0.61	0.62	0.29
Maximum deviation	5.03	1.62	8.12	1.47	2.09	0.58
Minimum deviation	−1.06	−1.16	1.42	−1.91	−1.18	−0.89
Absolute maximum	5.03	1.62	8.12	1.91	2.09	0.89

The most prominent feature in Figure 2 is the well‐known fact that HRPA significantly overestimates excitation energies (on average 4.03 eV compared to CC3).^21^, ^22^ Furthermore, RPA and HRPA exhibit much larger maximum deviations than all other methods and consequently the standard deviations of RPA and HRPA are larger than for the other methods. Also RPA overestimates on average the CC3 singlet excitation energies by 0.84 eV. Adding the noniterative doubles corrections (D) to both the RPA and HRPA singlet excitation energies completely changes the picture; all of the statistical measures are significantly reduced.

The pattern of the statistical data for the new HRPA(D) method resembles those of SOPPA. Both underestimate on average the CC3 results, SOPPA by 0.42 eV and HRPA(D) by 0.46 eV, though SOPPA has a significantly smaller standard deviation, 0.29 eV, which is the smallest for all the methods studied here, compared to 0.61 eV for HRPA(D). Also the maximum deviation is in HRPA(D) clearly larger than that in SOPPA. Nevertheless HRPA(D) appears to give reasonable approximations to SOPPA excitation energies. The addition of the extra term in s‐HRPA(D) shifts the errors slightly upward thereby reducing the mean deviation from CC3 results. This is as expected because the elements of **S**^{2}^ are primarily negative making the correction in eq. (31) positive.

The pattern of the statistical data in Figure 2 turns out to be similar in RPA(D) and s‐HRPA(D) and different from that for HRPA(D) and SOPPA. The RPA(D) and s‐HRPA(D) methods are more spread around the CC3 results with the mean deviations being −0.1 and 0.22 eV, which is a large improvement over SOPPA. However, the standard deviations of the two models are around twice the one of SOPPA, that is, 0.54 and 0.62 eV, and the maximum deviations are also approximately twice as large.

The error distributions (white circles) in the case of RPA(D), HRPA(D), s‐HRPA(D), and SOPPA, shown in Figure 2, indicate that the errors fall into two groups: one large group with smaller errors and one small group with slightly larger errors. This splitting is related to the amount of double excitation character in a given excited state which explains why it is not observed in the case of RPA and HRPA where no double excitations are included. In the following, we will represent the amount of double excitation character with the single excitation weight in percent because when the double excitation character increases, the single excitation weight decreases. The single excitation weight is calculated as

(32) $$R^{\mathrm{ph}}S^{\left(0,2\right)}R^{\mathrm{ph}}-R^{\mathrm{hp}}S^{\left(0,2\right)*}R^{\mathrm{hp}}.$$

**Figure 3:**
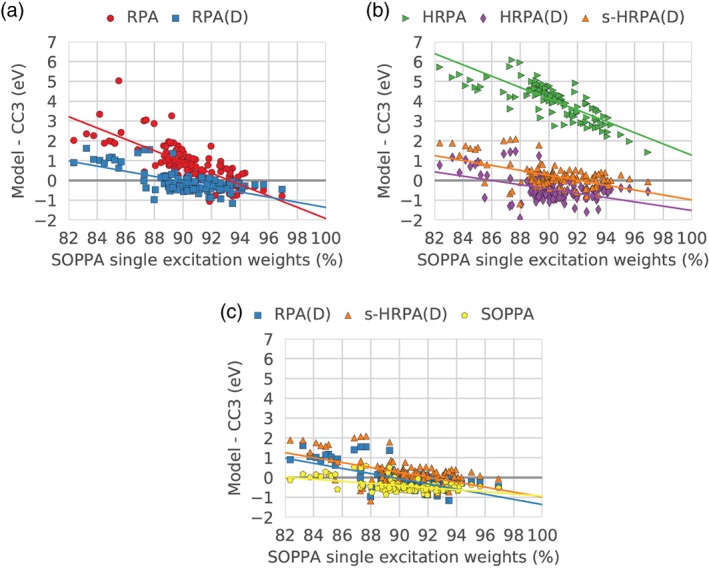
Correlation between the error relative to CC3 results with the different methods and the single excitation weight as calculated with SOPPA. The lines correspond to the best linear fit and are shown to guide the eye. [Color figure can be viewed at http://wileyonlinelibrary.com]

The correlation between the errors and the single excitation weights is illustrated in Figure 3, where the errors, again with respect to the CC3 results, are plotted against the single excitation weights (in percent) in the SOPPA calculations. We choose to compare to the SOPPA single excitation weights as these are obtained with the highest level of theory within this study.

The dominant observation from these figures is that there is a linear correlation between the deviation from the CC3 results and the single excitation weight. For smaller weights, all methods exhibit larger positive deviations. As the single excitation weight becomes larger the errors become firstly smaller and then negative with the exception of HRPA, where they always stay positive.

In more detail, Figure 3a shows the correlation of the RPA and RPA(D) errors. A clear trend is observed: the lower the single excitation weight, the higher the errors and the larger the difference between the RPA and RPA(D) errors. Including the double corrections in RPA(D) affects the excited states with lower single excitation weights the most, that is, the RPA and RPA(D) errors approach each other at higher single excitation weights, reflected in the two trend lines not being parallel.

The state with the largest RPA error (5.03 eV) is the ^3^B_1*g*_ state of s‐tetrazine. It has a low SOPPA single excitation weight (85.51%) but not among the lowest weights. In addition, a group of five states have RPA errors around 3 eV (2^1^B_2*g*_ and 2^1^B_1*g*_ of s‐tetrazine, 2^1^A′ of formamide, 4^1^B_2_ of pyridine, and 1^1^B_1*g*_ of pyrazine) but again the SOPPA single excitation weights (ranging from 84.17 to 89.21%) for these states are not the lowest of the set. The character of these six outliers from the general trend were investigated but no common features were found.

From Figure 2, it is clear that a group of states exhibits RPA(D) errors above 0.5 eV, significantly larger than the RPA(D) mean deviation of −0.10 eV. The SOPPA single excitation weights of these states range from 82.36 to 89.41% covering the lowest single excitation weights of the set.

Figure 3b shows the dependence of the HRPA errors on the SOPPA single excitation weights which is similar to that of RPA indicated by the similar slopes of the two trend lines (red in Fig. 3a and green in Fig. 3b). The HRPA errors are, however, shifted upwards with around 4 eV as seen also in Figure 2. The RPA outlier also shows up in HRPA with an error of 8.12 eV. For clarity, the plots have been zoomed in and this point is not visible in Figure 3. The (D) correction corrects both the large overestimation but also decreases the dependence on the SOPPA single excitation weight in a similar fashion as for the (D) correction to RPA (again seen by the similar slopes of the two trend lines; blue in Fig. 3a and purple in Fig. 3b). The effect of the different contributions of the (D) correction on the RPA and HRPA errors will be studied in more detail in a later section.

Adding the extra term to the HRPA(D) energies (s‐HRPA(D)) shifts the energies up but does not really change the dependency of the error on the single excitation weights (the two trend lines are almost parallel).

In Figure 3c, the three methods RPA(D), s‐HRPA(D), and SOPPA are compared, showing, as expected, that the two double‐corrected methods are more sensitive to the single excitation weight although already significantly less dependent than RPA and HRPA. At high single excitation weights, the RPA(D) and s‐HRPA(D) trend lines intersect the SOPPA trend line indicating that the performance of these three methods is similar as long as the single excitation weights are high. As previously noted, the dependency of the RPA(D) and s‐HRPA(D) errors on the single excitation weight is very similar (almost parallel trend lines) but the s‐HRPA(D) energies are in general slightly larger (on average 0.32 eV).

For this data set and all methods, it seems likely that the most reliable results are obtained, if the double excitation weight does not exceed 10*%*.

### Triplet excited states

Turning now to the excited triplet states, we present RPA(D) results for the first time here along with the two new methods HRPA(D) and s‐HRPA(D). As in the case of the excited singlet states, we have employed also the well‐known RPA and HRPA methods and included the SOPPA results from Ref. 27 for comparison.

**Figure 4:**
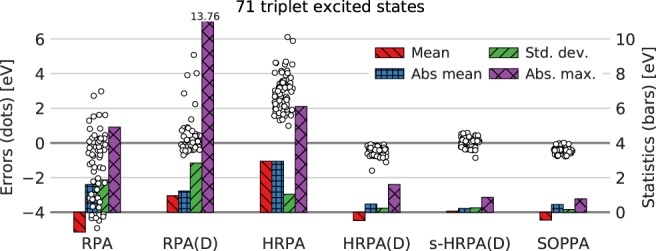
Distributions of errors (circles, left axis) relative to CC3 results (calculated as method—CC3) and statistical measures (bars, right axis) of 71 triplet excited states. Numerical values for statistical measures can be found in Supporting Information Table 2. TZVP basis set, frozen core. [Color figure can be viewed at http://wileyonlinelibrary.com]

In Figure 4, the distribution of errors with respect to CC3 values and corresponding statistical measures for the 71 triplet excited states are shown. Compared to the case of excited singlet states, Figure 2, the RPA and RPA(D) errors differ significantly. Whereas the RPA errors seem more spread out with a significant fraction being very negative, the RPA(D) method suffers from a small group of states with very large positive errors. For clarity Figure 4 has been zoomed in leaving out three RPA(D) states with errors of 8.8, 13.58, and 13.76 eV.

The large negative RPA errors are partly due to 16 states which have imaginary RPA excitation energies, that is, the real part is 0.0 eV and the error is thus equal to the negative of the value of the CC3 result. The same behavior was observed in the TDDFT study from Peach et al.^7^ upon inclusion of a large amount of exact (Hartree–Fock) exchange in DFT hybrid functionals for the 1^3^B_*u*_ state of butadiene, the 1^3^B_1*u*_ state of benzoquinone as well as the 1^3^B_2*u*_ state of naphthalene. In a recent study by Yang et al.,^30^ another seven imaginary triplet RPA excitation energies were reported. In addition to the 10 previously reported unstable RPA states, we observed another six imaginary excitation energies. The 16 states with imaginary excitation energies have several things in common: they are all of *π* → π* character; they are the first excited triplet states in a given symmetry; and they have relatively low CC3 excitation energies in the range 2.30–4.48 eV and relatively high SOPPA single excitation weights in the range 93.94–97.97% (the majority around 96%). They have been marked with gray in Supporting Information Table S5, and in Table 2, the statistical analysis is shown with (71 states) and without (55 states) these states for the RPA and RPA(D) methods.

**Table 2:** Statistical measures (eV) of 71 triplet excited states.

		RPA			RPA(D)		HRPA	HRPA(D)	s‐HRPA(D)	SOPPA
Count	71	55	50	71	55	50	71	71	71	71
Mean	−1.14	−0.41	−0.10	0.96	0.43	−0.07	2.95	−0.48	0.07	−0.45
Absolute mean	1.60	1.00	0.75	1.23	0.74	0.27	2.95	0.48	0.22	0.45
Standard deviation	1.87	1.43	1.07	2.84	2.04	0.34	1.05	0.24	0.26	0.16
Maximum deviation	2.98	2.98	2.98	13.76	13.76	0.90	6.11	−0.08	0.58	0.01
Minimum deviation	−4.92	−4.92	−2.34	−0.70	−0.70	−0.70	0.99	−1.60	−0.86	−0.78
Absolute maximum	4.92	4.92	2.98	13.76	13.76	0.90	6.11	1.60	0.86	0.78

Because the RPA(D) method adds a noniterative correction to the RPA excitation energy, the RPA(D) results for these states should be meaningless which in some cases results in the large overestimation seen in Figure 4. This behavior is due to the noniterative correction making use of the RPA eigenvectors which in the case of imaginary RPA excitation energies are meaningless. In addition, the five RPA(D) results with large positive errors (above and well above 2 eV) in Figure 4 indicate another group of unstable RPA results within the remaining 55 states which has been marked with a dark gray color in Supporting Information Table S5. This group have large negative RPA errors between −3.01 and − 4.92 eV and share the same characteristics as the states with imaginary RPA energies, see previous paragraph. In Refs. 7 and 30 such behavior was observed as well, that is, large underestimation of triplet excitation energies upon inclusion of large amount of exact exchange or by using the RPA method, respectively. In Table 2 also these five states have been excluded from the RPA and RPA(D) results leaving 50 states which seem to behave normally and which results in statistical measures comparable to those of the singlet excited states. The remaining discussion is based on the set of 50 states in the case of the RPA and RPA(D) methods and the full set of 71 states for the remaining methods as the effect of considering only the 50 states on the statistical measures of the remaining methods is negligible as shown in Supporting Information Table S1. The exclusion of unstable states without imaginary RPA excitation energies is rather qualitative and only possible due to the existence of reference values and a more rigorous stability criterion would be preferable as discussed in Ref. 7.

This behavior indicates an unstable eigenvalue problem related to the well‐known problem of triplet instabilities^28^ as was also discussed in Ref. 7. A triplet instability is associated with the existence of a spin‐unrestricted Hartree–Fock solution with lower energy compared to the employed restricted Hartree–Fock solution. In Ref. 7, it was shown that the problem of triplet instabilities could be greatly reduced when employing the TDA and analogously the CIS results of Ref. 30 show no problems with triplet instabilities. The problem of triplet instabilities can of course be drastically reduced by using a method which includes more correlation as indicated by the SOPPA results presented in Ref. 27.

**Figure 5:**
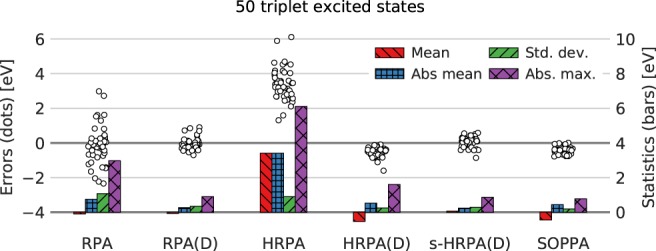
Distributions of errors (circles, left axis) relative to CC3 results (calculated as method—CC3) and statistical measures (bars, right axis) of 50 triplet excited states. Numerical values for statistical measures can be found in Supporting Information Table 2. TZVP basis set, frozen core. [Color figure can be viewed at http://wileyonlinelibrary.com]

In Figure 5, the distributions and statistical measures are shown for the 50 states. Both the RPA and RPA(D) results seem to be distributed around the CC3 results. This behavior is different from the singlet case where the RPA method overestimated the energies with around 1 eV on average. Two states are considerably overestimated with the RPA method with errors around 3 eV. As in the case of the excited singlet states, these two states have some of the lowest single excitation weights of the set, see later discussion.

The HRPA method overestimates the triplet excitation energies slightly less than the singlet excitation energies with the mean deviations being 2.95 and 4.03 eV while the standard deviations in both cases are very similar. An advantageous feature of the HRPA method is that it is seemingly unaffected by (triplet) instabilities, as also suggested in Ref. 53, indicated by similar statistical measures when excluding the unstable states as shown in Supporting Information Table S1. The HRPA(D) method again provides a significant improvement of the HRPA results and the s‐HRPA(D) centers the energies around the CC3 results. Consequently, if one studies a system where RPA is (or might be) unstable and wants to avoid the rather qualitative exclusion of unstable states with the RPA and RPA(D) methods, the HRPA(D) and in particular the s‐HRPA(D) methods are superior to the RPA(D) method for excited triplet states.

Again, the statistical measures of the HRPA(D) errors resemble those of the SOPPA errors whereas the s‐HRPA(D) results are closer to the CC3 results. The RPA(D) and s‐HRPA(D) methods result in the smallest mean deviations of −0.07 and 0.07 eV compared to −0.52 for HRPA(D) and –0.44 for SOPPA. The smallest standard deviation of the errors is again obtained with the SOPPA method (0.18 eV) with the HRPA(D) and s‐HRPA(D) standard deviations being around 1.5 times as large (0.25 and 0.28 eV) and the RPA(D) almost double as large (0.34 eV).

A comparison of the excited singlet and triplet states, Figures 2 and 5, leads to the impression that all methods perform better for triplet excitation energies. For example, the standard deviations of the HRPA(D) and s‐HRPA(D) errors for singlet excitation energies are more than twice the standard deviations of the triplet excitation energies. However, one has to be careful when comparing the sets of excited singlet and triplet states as the single excitation weights are in general higher for the triplet excitation energies than for the singlet excitation energies. In the discussion of the excited singlet states, it was found that higher single excitation weights resulted in lower errors. The single excitation weights for the excited triplet states are in the range 87–98%, whereas the excited singlet states have single excitation weights of 82–97%.

**Figure 6:**
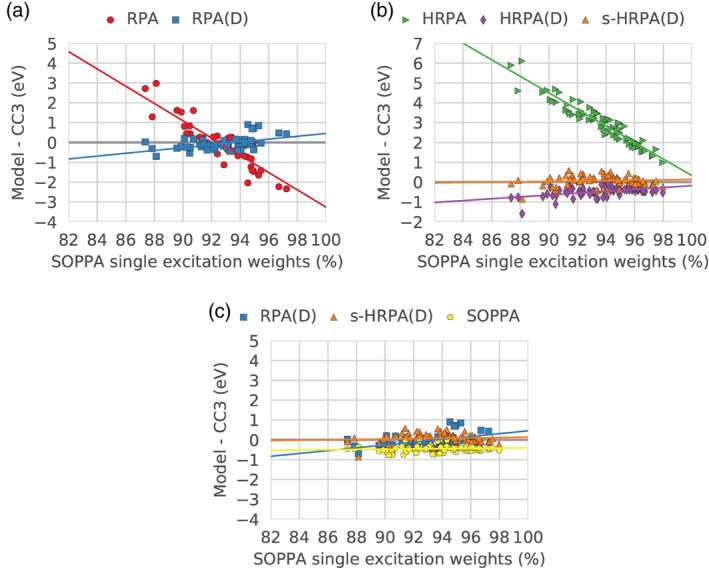
Correlation between the error relative to CC3 results with the different methods and the single excitation weight as calculated with SOPPA. [Color figure can be viewed at http://wileyonlinelibrary.com]

The dependence of the errors of the excited triplet states on the amount of single excitation weight in the SOPPA calculations is illustrated in Figure 6. Again, there is a clear linear dependence of the errors on the single excitation weight.

As expected, the RPA and HRPA errors in Figures 6a and 6b show the strongest dependence on the single excitation weights. This dependency is greatly reduced by the inclusion of double corrections. Whereas the RPA(D) and HRPA(D) methods resulted in errors larger than the mean deviation at lower single excitation weights for excited singlet states, the two methods show the opposite trend for excited triplet states, that is, errors lower than the mean deviation at low single excitation weights. In other words, the slopes of the (H)RPA and (H)RPA(D) trend lines have different sign in the triplet case but same sign in the singlet case. In the case of the excited singlet states the HRPA(D) and s‐HRPA(D) trend lines were close to parallel which is not the case for the excited triplet states, Figure 6b. Except for one outlier at low single excitation weight, the s‐HRPA(D) method shows no dependence on the amount of single excitation weight.

Analogous to the singlet case, the largest positive RPA errors (>0.5 eV) correspond to the lowest SOPPA single excitation weights (around 90% or lower except for one which is 92.4%). The two largest positive RPA errors have SOPPA single excitation weights of 87.4 and 88.1% and correspond to the 9th and 10th excited triplet state of the s‐tetrazine molecule of *n* → π* character. The same two states also cause the largest HRPA errors and the latter results in the largest negative RPA(D), HRPA(D), and s‐HRPA(D) errors of −0.7, −1.6 and –0.86 eV.

Finally, in Figure 6c, the three best methods RPA(D), s‐HRPA(D), and SOPPA are compared. The majority of the RPA(D) errors lies somewhere between the SOPPA and s‐HRPA(D) errors. Both the s‐HRPA(D) and SOPPA errors show no significant dependence on the single excitation weights. This is different from the singlet case where a larger dependence on the single excitation weights was observed, but one should again remember that the single excitation weights are generally higher for the set of triplet states.

### Second‐order double correction contributions

**Figure 7:**
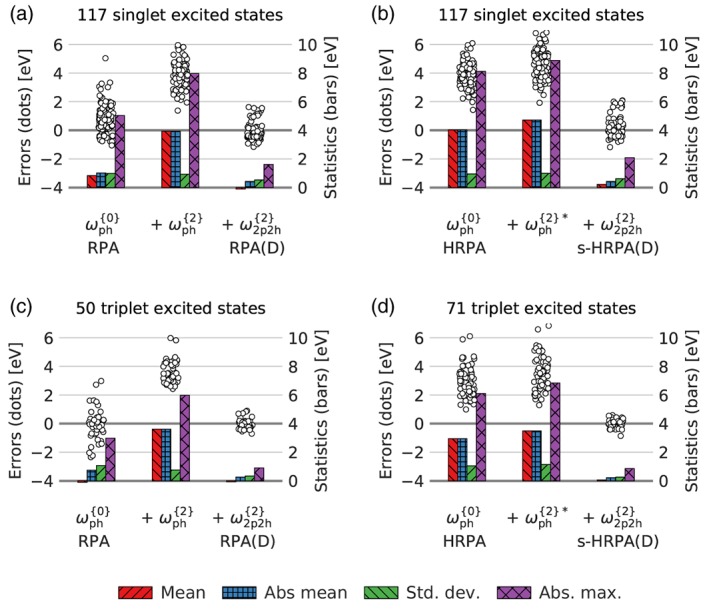
Distribution of errors (dots) and statistical measures (bars) of the different types of doubles corrections in the RPA(D) (left) and s‐HRPA(D) (right) methods for singlet (top) and triplet (bottom) excitation energies. [Color figure can be viewed at http://wileyonlinelibrary.com]

Finally, we will investigate the effect of the individual noniterative contributions in the RPA(D) and s‐HRPA(D) methods. In Figure 7, the errors of the individual contributions of the noniterative second‐order doubles corrections are shown for the singlet and triplet results.

In the case of RPA(D), there are two types of contributions in addition to the RPA excitation energy: the second‐order contribution to the particle–hole part, $\omega_{j,\mathrm{ph}}^{\left\{2\right\}}$, eq. (24), and the second‐order contribution to the two‐particle–two‐hole part, $\omega_{j,2p2h}^{\left\{2\right\}}$, eq. (25). These are shown for excited singlet and triplet states in the left part of Figure 7. The first contribution, $\omega_{j,\mathrm{ph}}^{\left\{2\right\}}$, shifts the errors up by a few eV. In the singlet case, the distribution of the errors remains seemingly unchanged whereas in the triplet case the spread of the errors is decreased. This makes in particular the singlet results resemble those of the HRPA method as expected as the $\omega_{j,\mathrm{ph}}^{\left\{2\right\}}$ correction is just the noniterative form of the HRPA formalism. In both the singlet and triplet cases, the noniterative second‐order doubles correction $\omega_{j,2p2h}^{\left\{2\right\}}$ reduces both the mean and standard deviations considerably.

In the case of s‐HRPA(D), the $\omega_{j,\mathrm{ph}}^{\left\{2\right\}*}$ correction, eq. 31, is different from the RPA(D) case because the second‐order correction to the particle–hole part is already included in the HRPA method. The effect of the $\omega_{j,\mathrm{ph}}^{\left\{2\right\}*}$ correction to the HRPA singlet and triplet excitation energies, right side of Figure 7, is thus significantly smaller than the $\omega_{j,\mathrm{ph}}^{\left\{2\right\}}$ correction to RPA. The $\omega_{j,\mathrm{ph}}^{\left\{2\right\}*}$ correction shifts the errors to slightly larger values leaving the standard deviation unchanged, which is now clear for both the singlet and triplet excitation energies, and the $\omega_{j,2p2h}^{\left\{2\right\}}$ correction has the same effect as in the case of RPA(D).

### Timings

Before concluding, we need to briefly discuss the timings of the new methods. In Table 3 the total wall time of the RPA(D) and HRPA(D) calculations in percent of the wall time of the SOPPA calculation is illustrated for two test systems, benzene and naphthalene, and for both singlet and triplet excitation energies. The s‐HRPA(D) method is not included here as the time consumption is very similar to the HRPA(D) method. The corresponding absolute wall times are shown in Supporting Information Table S8.

**Table 3:** Wall time in percent of SOPPA wall time for the RPA(D) and HRPA(D) methods.

		RPA(D)	HRPA(D)
Benzene	Singlet	13.04	57.17
	Triplet	13.96	44.93
Naphthalene	Singlet	11.50	57.25
	Triplet	12.59	45.43

As expected, the saving in computation time is greater with the RPA(D) method than with the HRPA(D) method. The most expensive part of a SOPPA calculation is the linear transformation of a trial vector with the **B**^(2)^ matrix which scales as *N* ^4^ *O* with *N* being the number of basis functions and *O* the number of occupied orbitals. In SOPPA and HRPA(D), this term is evaluated in each iteration, whereas the terms ignored in HRPA(D) scales at most as *N* ^3^ *O* ^2^. Thus, the moderate saving in computation time of HRPA(D) comes as much from the faster convergence of HRPA(D) calculations as it comes from a lower cost per iteration, when compared to SOPPA calculations. The faster convergence of HRPA(D) compared to SOPPA is due to the large double excitation manifold which is included in SOPPA and which makes convergence slower. In RPA(D), the transformation of the converged RPA eigenvector with the **B**^(2)^ matrix is evaluated only once after convergence of the RPA equations, which scale as *N* ^4^, making the savings considerably larger.

Whereas the wall time of the RPA(D) calculations is around 12–14% of the SOPPA wall time, the wall time of the HRPA(D) calculation varies from being ~57% for singlet excitation energies to ~45% for triplet excitation energies compared to the SOPPA wall time. This difference probably originates from the fact that the triplet double excitation operator, in the spin‐adapted basis used here, consists of roughly 50% more independent parameters which increases the workload on the calculations of the **C** and **D** matrices compared to the singlet case. Consequently, the noniterative treatment of these matrices in the HRPA(D) method results in a slightly larger saving in the case of triplet excitation energies compared to singlet excitation energies.

## Conclusions

Two new methods, HRPA(D) and s‐HRPA(D), have been presented for calculating singlet and triplet excitation energies. Furthermore, the RPA(D) method has been extended to triplet excited states in addition to the existing singlet implementation.^23^ The performance of the second‐order doubles‐corrected methods has been compared to the SOPPA and CC3 methods by using a large benchmark set consisting of 117 singlet and 71 triplet excitation energies.

The double corrections to RPA and HRPA both reduce the mean as well as the standard deviation of the errors significantly. The effect of the doubles correction is largest for states with the highest double excitation weight. Of the methods tested here, the standard deviation of the errors compared to the CC3 results is smallest with SOPPA. The standard deviations of the doubles‐corrected methods are around twice that of SOPPA. Whereas SOPPA and HRPA(D) underestimate excitation energies with ~0.5 eV, the RPA(D) shows the smallest mean deviation for singlet excitation energies, −0.1 eV, and s‐HRPA(D) shows the smallest for triplet excitation energies, 0.07 eV. The shift term included in the s‐HRPA(D) method thus results in a slight increase of excitation energies, which decreases the deviation from CC3 results compared to HRPA(D) and SOPPA.

An advantage of the HRPA(D) method compared to the RPA(D) method is that it is seemingly unaffected by triplet instabilities. Triplet instabilities are well known to occur when using the RPA method. In the worst case, an RPA excitation energy can become imaginary and the eigenvector has no physical meaning. The RPA(D) energy will then also be meaningless, as the RPA(D) method uses both the RPA energies and eigenvectors to construct the noniterative correction.

To summarize, while the errors of the RPA(D), HRPA(D), and s‐HRPA(D) methods are similar, the HRPA(D) and s‐HRPA(D) methods do not suffer from triplet instabilities. On the other hand the savings in computation time are much larger and more consistent with the RPA(D) method, as also observed in Ref. 42. The implementation of triplet excitation energies with the doubles corrected methods enabled the further implementation of nuclear spin–spin coupling constants which is also presented in Ref. 42.

## Supporting information


**Appendix S1**. Supporting Information.
