# Supplementary material for: Noniterative Doubles Corrections to the Random Phase and Higher Random Phase Approximations: Singlet and Triplet Excitation Energies
Source: J Comput Chem. 2019 Oct 1;41(1):43–55. doi: 10.1002/jcc.26074 (PMC6899555; doi:10.1002/jcc.26074)
Supplement: Supplementary file 1 — Appendix S1. Supporting Information. [file JCC-41-43-s001.pdf]

# Supplementary information: Non-iterative doubles corrections to the random phase and higher random phase approximations: singlet and triplet excitation energies

Pi A. B. Haase, Rasmus Faber, Patricio F. Provasi, Stephan P. A. Sauer

## 1 Supplementary information

### 1.1 Statistical measures when excluding unstable states

| | HRPA | | | HRPA(D) | | | s-HRPA(D) | | | SOPPA | | |
|---|---|---|---|---|---|---|---|---|---|---|---|---|
| Count | 71 | 55 | 50 | 71 | 55 | 50 | 71 | 55 | 50 | 71 | 55 | 50 |
| Mean | 2.95 | 3.27 | 3.41 | -0.48 | -0.52 | -0.53 | 0.07 | 0.07 | 0.07 | -0.45 | -0.44 | -0.44 |
| Abs mean | 2.95 | 3.27 | 3.41 | 0.48 | 0.52 | 0.53 | 0.22 | 0.23 | 0.24 | 0.45 | 0.44 | 0.44 |
| Std dev | 1.05 | 0.96 | 0.9 | 0.24 | 0.25 | 0.25 | 0.26 | 0.28 | 0.29 | 0.16 | 0.18 | 0.19 |
| Max dev | 6.11 | 6.11 | 6.11 | -0.08 | -0.09 | -0.09 | 0.58 | 0.58 | 0.58 | 0.01 | 0.01 | 0.01 |
| Min dev | 0.99 | 1.31 | 1.31 | -1.6 | -1.6 | -1.6 | -0.86 | -0.86 | -0.86 | -0.78 | -0.78 | -0.78 |
| Abs max | 6.11 | 6.11 | 6.11 | 1.6 | 1.6 | 1.6 | 0.86 | 0.86 | 0.86 | 0.78 | 0.78 | 0.78 |

Table 1: Statistical measures (eV) of the errors with respect to CC3 results of triplet excitation energies when including 71 (all), 55 (without imaginary RPA results) and 50 (without large RPA errors).

## 1.2 117 Singlet excited states: Excited state information

Table 2: Excited state characteristics of 117 singlet excited states.

| Nr. | Molecule | Point group | State | | |
|---|---|---|---|---|---|
| 1 | Ethene | $D_{2h}$ | 1 | $^1B_{1u}$ | $(\pi \rightarrow \pi^*)$ |
| 2 | E-Butadiene | $C_{2h}$ | 1 | $^1B_u$ | $(\pi \rightarrow \pi^*)$ |
| 3 | E-Butadiene | $C_{2h}$ | 1 | $^1A_g$ | $(\pi \rightarrow \pi^*)$ |
| 4 | Hexatriene | $C_{2h}$ | 1 | $^1B_u$ | $(\pi \rightarrow \pi^*)$ |
| 5 | Hexatriene | $C_{2h}$ | 1 | $^1A_g$ | $(\pi \rightarrow \pi^*)$ |
| 6 | Octatetraene | $C_{2h}$ | 1 | $^1A_g$ | $(\pi \rightarrow \pi^*)$ |
| 7 | Octatetraene | $C_{2h}$ | 1 | $^1B_u$ | $(\pi \rightarrow \pi^*)$ |
| 8 | Octatetraene | $C_{2h}$ | 2 | $^1B_u$ | $(\pi \rightarrow \pi^*)$ |
| 9 | Octatetraene | $C_{2h}$ | $2^a$ | $^1A_g$ | $(\pi \rightarrow \pi^*)$ |
| 10 | Octatetraene | $C_{2h}$ | $3^b$ | $^1A_g$ | $(\pi \rightarrow \pi^*)$ |
| 11 | Octatetraene | $C_{2h}$ | 3 | $^1B_u$ | $(\pi \rightarrow \pi^*)$ |
| 12 | Cyclopropene | $C_{2v}$ | 1 | $^1B_1$ | $(\sigma \rightarrow \pi^*)$ |
| 13 | Cyclopropene | $C_{2v}$ | 1 | $^1B_2$ | $(\pi \rightarrow \pi^*)$ |
| 14 | Cyclopentadiene | $C_{2v}$ | 1 | $^1B_2$ | $(\pi \rightarrow \pi^*)$ |
| 15 | Cyclopentadiene | $C_{2v}$ | $1^c$ | $^1A_1$ | $(\pi \rightarrow \pi^*)$ |
| 16 | Cyclopentadiene | $C_{2v}$ | $2^d$ | $^1A_1$ | $(\pi \rightarrow \pi^*)$ |
| 17 | Norbornadiene | $C_{2v}$ | 1 | $^1A_2$ | $(\pi \rightarrow \pi^*)$ |
| 18 | Norbornadiene | $C_{2v}$ | 1 | $^1B_2$ | $(\pi \rightarrow \pi^*)$ |
| 19 | Norbornadiene | $C_{2v}$ | 2 | $^1B_2$ | $(\pi \rightarrow \pi^*)$ |
| 20 | Norbornadiene | $C_{2v}$ | 2 | $^1A_2$ | $(\pi \rightarrow \pi^*)$ |
| 21 | Benzene | $D_{2h}$ | 1 | $^1B_{3u}$ | $(\pi \rightarrow \pi^*)$ |
| 22 | Benzene | $D_{2h}$ | 1 | $^1B_{2u}$ | $(\pi \rightarrow \pi^*)$ |
| 23 | Benzene | $D_{2h}$ | 2 | $^1B_{3u}$ | $(\pi \rightarrow \pi^*)$ |
| 24 | Benzene | $D_{2h}$ | 1 | $^1A_g$ | $(\pi \rightarrow \pi^*)$ |
| 25 | Naphthalene | $D_{2h}$ | 1 | $^1B_{3u}$ | $(\pi \rightarrow \pi^*)$ |
| 26 | Naphthalene | $D_{2h}$ | 1 | $^1B_{2u}$ | $(\pi \rightarrow \pi^*)$ |
| 27 | Naphthalene | $D_{2h}$ | 1 | $^1A_g$ | $(\pi \rightarrow \pi^*)$ |
| 28 | Naphthalene | $D_{2h}$ | 1 | $^1B_{1g}$ | $(\pi \rightarrow \pi^*)$ |
| 29 | Naphthalene | $D_{2h}$ | 2 | $^1B_{3u}$ | $(\pi \rightarrow \pi^*)$ |
| 30 | Naphthalene | $D_{2h}$ | 2 | $^1B_{2u}$ | $(\pi \rightarrow \pi^*)$ |
| 31 | Naphthalene | $D_{2h}$ | 2 | $^1B_{1g}$ | $(\pi \rightarrow \pi^*)$ |
| 32 | Naphthalene | $D_{2h}$ | 2 | $^1A_g$ | $(\pi \rightarrow \pi^*)$ |
| 33 | Naphthalene | $D_{2h}$ | 3 | $^1B_{3u}$ | $(\pi \rightarrow \pi^*)$ |
| 34 | Naphthalene | $D_{2h}$ | 3 | $^1B_{2u}$ | $(\pi \rightarrow \pi^*)$ |
| 35 | Furan | $C_{2v}$ | 1 | $^1B_2$ | $(\pi \rightarrow \pi^*)$ |
| 36 | Furan | $C_{2v}$ | 1 | $^1A_1$ | $(\pi \rightarrow \pi^*)$ |
| 37 | Furan | $C_{2v}$ | 2 | $^1A_1$ | $(\pi \rightarrow \pi^*)$ |

Table 2: Excited state characteristics of 117 singlet excited states.

| Nr. | Molecule | Point group | State | | |
|---|---|---|---|---|---|
| 38 | Pyrrole | $C_{2v}$ | 1 | $^1A_1$ | $(\pi \rightarrow \pi^*)$ |
| 39 | Pyrrole | $C_{2v}$ | 1 | $^1B_2$ | $(\pi \rightarrow \pi^*)$ |
| 40 | Pyrrole | $C_{2v}$ | 2 | $^1A_1$ | $(\pi \rightarrow \pi^*)$ |
| 41 | Imidazole | $C_s$ | $1^e$ | $^1A'$ | $(\pi \rightarrow \pi^*)$ |
| 42 | Imidazole | $C_s$ | $1^f$ | $^1A$" | $(n \rightarrow \pi^*)$ |
| 43 | Imidazole | $C_s$ | $2^g$ | $^1A'$ | $(\pi \rightarrow \pi^*)$ |
| 44 | Imidazole | $C_s$ | $3^h$ | $^1A$" | $(n \rightarrow \pi^*)$ |
| 45 | Imidazole | $C_s$ | $4^i$ | $^1A'$ | $(\pi \rightarrow \pi^*)$ |
| 46 | Pyridine | $C_{2v}$ | 1 | $^1B_2$ | $(\pi \rightarrow \pi^*)$ |
| 47 | Pyridine | $C_{2v}$ | 1 | $^1B_1$ | $(n \rightarrow \pi^*)$ |
| 48 | Pyridine | $C_{2v}$ | 1 | $^1A_2$ | $(n \rightarrow \pi^*)$ |
| 49 | Pyridine | $C_{2v}$ | 1 | $^1A_1$ | $(\pi \rightarrow \pi^*)$ |
| 50 | Pyridine | $C_{2v}$ | 2 | $^1A_1$ | $(\pi \rightarrow \pi^*)$ |
| 51 | Pyridine | $C_{2v}$ | 2 | $^1B_2$ | $(\pi \rightarrow \pi^*)$ |
| 52 | Pyridine | $C_{2v}$ | $4^j$ | $^1B_2$ | $(\pi \rightarrow \pi^*)$ |
| 53 | Pyridine | $C_{2v}$ | $4^k$ | $^1A_1$ | $(\pi \rightarrow \pi^*)$ |
| 54 | Pyrazine | $D_{2h}$ | 1 | $^1B_{3u}$ | $(n \rightarrow \pi^*)$ |
| 55 | Pyrazine | $D_{2h}$ | 1 | $^1A_u$ | $(n \rightarrow \pi^*)$ |
| 56 | Pyrazine | $D_{2h}$ | 1 | $^1B_{2u}$ | $(\pi \rightarrow \pi^*)$ |
| 57 | Pyrazine | $D_{2h}$ | 1 | $^1B_{2g}$ | $(n \rightarrow \pi^*)$ |
| 58 | Pyrazine | $D_{2h}$ | $1^l$ | $^1B_{1g}$ | $(n \rightarrow \pi^*)$ |
| 59 | Pyrazine | $D_{2h}$ | 1 | $^1B_{1u}$ | $(\pi \rightarrow \pi^*)$ |
| 60 | Pyrazine | $D_{2h}$ | 2 | $^1B_{2u}$ | $(\pi \rightarrow \pi^*)$ |
| 61 | Pyrazine | $D_{2h}$ | 2 | $^1B_{1u}$ | $(\pi \rightarrow \pi^*)$ |
| 62 | Pyrazine | $D_{2h}$ | $1^m$ | $^1B_{3g}$ | $(\pi \rightarrow \pi^*)$ |
| 63 | Pyrazine | $D_{2h}$ | $3^n$ | $^1A_g$ | $(\pi \rightarrow \pi^*)$ |
| 64 | Pyrimidine | $C_{2v}$ | 1 | $^1B_1$ | $(n \rightarrow \pi^*)$ |
| 65 | Pyrimidine | $C_{2v}$ | 1 | $^1A_2$ | $(n \rightarrow \pi^*)$ |
| 66 | Pyrimidine | $C_{2v}$ | 1 | $^1B_2$ | $(\pi \rightarrow \pi^*)$ |
| 67 | Pyrimidine | $C_{2v}$ | 1 | $^1A_1$ | $(\pi \rightarrow \pi^*)$ |
| 68 | Pyrimidine | $C_{2v}$ | 2 | $^1A_1$ | $(\pi \rightarrow \pi^*)$ |
| 69 | Pyrimidine | $C_{2v}$ | 2 | $^1B_2$ | $(\pi \rightarrow \pi^*)$ |
| 70 | Pyridazine | $C_{2v}$ | 1 | $^1B_1$ | $(n \rightarrow \pi^*)$ |
| 71 | Pyridazine | $C_{2v}$ | 1 | $^1A_2$ | $(n \rightarrow \pi^*)$ |
| 72 | Pyridazine | $C_{2v}$ | 1 | $^1A_1$ | $(\pi \rightarrow \pi^*)$ |
| 73 | Pyridazine | $C_{2v}$ | 2 | $^1A_2$ | $(n \rightarrow \pi^*)$ |
| 74 | Pyridazine | $C_{2v}$ | 2 | $^1B_1$ | $(n \rightarrow \pi^*)$ |
| 75 | Pyridazine | $C_{2v}$ | 1 | $^1B_2$ | $(\pi \rightarrow \pi^*)$ |
| 76 | Pyridazine | $C_{2v}$ | 2 | $^1B_2$ | $(\pi \rightarrow \pi^*)$ |

Table 2: Excited state characteristics of 117 singlet excited states.

| Nr. | Molecule | Point group | State | | |
|---|---|---|---|---|---|
| 77 | Pyridazine | $C_{2v}$ | 2 | $^1A_1$ | $(\pi \rightarrow \pi^*)$ |
| 78 | s-Triazine | $C_{2v}$ | 1 | $^1A_2$ | $(n \rightarrow \pi^*)$ |
| 79 | s-Triazine | $C_{2v}$ | 2 | $^1B_1$ | $(n \rightarrow \pi^*)$ |
| 80 | s-Triazine | $C_{2v}$ | 1 | $^1B_1$ | $(n \rightarrow \pi^*)$ |
| 81 | s-Triazine | $C_{2v}$ | 1 | $^1B_2$ | $(\pi \rightarrow \pi^*)$ |
| 82 | s-Triazine | $C_{2v}$ | 1 | $^1A_1$ | $(\pi \rightarrow \pi^*)$ |
| 83 | s-Triazine | $C_{2v}$ | 3 | $^1B_1$ | $(n \rightarrow \pi^*)$ |
| 84 | s-Triazine | $C_{2v}$ | 2 | $^1A_1$ | $(\pi \rightarrow \pi^*)$ |
| 85 | s-Tetrazine | $D_{2h}$ | 1 | $^1B_{3u}$ | $(n \rightarrow \pi^*)$ |
| 86 | s-Tetrazine | $D_{2h}$ | 1 | $^1A_u$ | $(\pi \rightarrow \pi^*)$ |
| 87 | s-Tetrazine | $D_{2h}$ | 1 | $^1B_{1g}$ | $(n \rightarrow \pi^*)$ |
| 88 | s-Tetrazine | $D_{2h}$ | 1 | $^1B_{2u}$ | $(\pi \rightarrow \pi^*)$ |
| 89 | s-Tetrazine | $D_{2h}$ | 1 | $^1B_{2g}$ | $(n \rightarrow \pi^*)$ |
| 90 | s-Tetrazine | $D_{2h}$ | 2 | $^1A_u$ | $(n \rightarrow \pi^*)$ |
| 91 | s-Tetrazine | $D_{2h}$ | 2 | $^1B_{2g}$ | $(n \rightarrow \pi^*)$ |
| 92 | s-Tetrazine | $D_{2h}$ | 2 | $^1B_{1g}$ | $(n \rightarrow \pi^*)$ |
| 93 | s-Tetrazine | $D_{2h}$ | $3^o$ | $^1B_{1g}$ | $(n \rightarrow \pi^*)$ |
| 94 | s-Tetrazine | $D_{2h}$ | 2 | $^1B_{3u}$ | $(n \rightarrow \pi^*)$ |
| 95 | s-Tetrazine | $D_{2h}$ | $1^p$ | $^1B_{1u}$ | $(\pi \rightarrow \pi^*)$ |
| 96 | s-Tetrazine | $D_{2h}$ | $2^q$ | $^1B_{1u}$ | $(\pi \rightarrow \pi^*)$ |
| 97 | s-Tetrazine | $D_{2h}$ | 2 | $^1B_{2u}$ | $(\pi \rightarrow \pi^*)$ |
| 98 | s-Tetrazine | $D_{2h}$ | $2^r$ | $^1B_{3g}$ | $(\pi \rightarrow \pi^*)$ |
| 99 | Formaldehyde | $C_{2v}$ | 1 | $^1A_2$ | $(n \rightarrow \pi^*)$ |
| 100 | Formaldehyde | $C_{2v}$ | 1 | $^1B_2$ | $(\sigma \rightarrow \pi^*)$ |
| 101 | Formaldehyde | $C_{2v}$ | 2 | $^1A_1$ | $(\sigma \rightarrow \pi^*)$ |
| 102 | Acetone | $C_{2v}$ | 1 | $^1A_2$ | $(n \rightarrow \pi^*)$ |
| 103 | Acetone | $C_{2v}$ | 1 | $^1B_2$ | $(\sigma \rightarrow \pi^*)$ |
| 104 | Acetone | $C_{2v}$ | $2^s$ | $^1A_1$ | $(\pi \rightarrow \pi^*)$ |
| 105 | p-Benzoquinone | $D_{2h}$ | 1 | $^1B_{1g}$ | $(n \rightarrow \pi^*)$ |
| 106 | p-Benzoquinone | $D_{2h}$ | 1 | $^1A_u$ | $(n \rightarrow \pi^*)$ |
| 107 | p-Benzoquinone | $D_{2h}$ | 1 | $^1B_{3g}$ | $(\pi \rightarrow \pi^*)$ |
| 108 | p-Benzoquinone | $D_{2h}$ | 1 | $^1B_{1u}$ | $(\pi \rightarrow \pi^*)$ |
| 109 | p-Benzoquinone | $D_{2h}$ | 1 | $^1B_{3u}$ | $(n \rightarrow \pi^*)$ |
| 110 | p-Benzoquinone | $D_{2h}$ | 2 | $^1B_{3g}$ | $(\pi \rightarrow \pi^*)$ |
| 111 | p-Benzoquinone | $D_{2h}$ | 2 | $^1B_{1u}$ | $(\pi \rightarrow \pi^*)$ |
| 112 | Formamide | $C_s$ | 1 | $^1A$" | $(n \rightarrow \pi^*)$ |
| 113 | Formamide | $C_s$ | 2 | $^1A'$ | $(\pi \rightarrow \pi^*)$ |
| 114 | Acetamide | $C_s$ | 1 | $^1A$" | $(n \rightarrow \pi^*)$ |
| 115 | Acetamide | $C_s$ | $2^t$ | $^1A'$ | $(\pi \rightarrow \pi^*)$ |

Table 2: Excited state characteristics of 117 singlet excited states.

| Nr. | Molecule | Point group | State | | |
|---|---|---|---|---|---|
| 116 | Propanamide | $C_s$ | 1 | $^1A$" | $(n \rightarrow \pi^*)$ |
| 117 | Propanamide | $C_s$ | $2^u$ | $^1A'$ | $(\pi \rightarrow \pi^*)$ |

[a] HRPA state nr. 3

[b] HRPA state nr. 2

[c] HRPA state nr. 2

[d] HRPA state nr. 1

[e] RPA and HRPA state nr. 2

[f] RPA state nr. 2 and HRPA state nr. 3

[g] RPA and HRPA state nr. 1

[h] RPA state nr. 4 and HRPA state nr. 5

[i] RPA and HRPA states nr. 3

[j] RPA state nr. 5

[k] RPA state nr. 3

[l] RPA and HRPA states nr. 3

[m] HRPA state nr. 2

[n] RPA state nr. 2

[o] RPA state nr. 4 and HRPA state nr. 5

[p] HRPA state nr. 2

[q] HRPA state nr. 1

[r] HRPA state nr. 3

[s] RPA and HRPA states nr. 1

[t] RPA state nr. 1

[u] RPA state nr. 1

## 1.3 117 singlet excited states: Excitation energies

Table 3: Excitation energies [eV] of 117 singlet excited states on different levels of theory. SOPPA results are taken from S. P. A. Sauer et al., Mol. Phys. 113, 2026 (2015).

| Nr. | molecule | RPA | RPA(D) | HRPA | HRPA(D) | s-HRPA(D) | SOPPA |
|---|---|---|---|---|---|---|---|
| 1 | Ethene | 7.58 | 7.92 | 9.79 | 7.81 | 8.30 | 7.84 |
| 2 | E-Butadiene | 6.08 | 6.12 | 8.80 | 5.98 | 6.56 | 5.89 |
| 3 | E-Butadiene | 8.02 | 8.15 | 10.37 | 8.02 | 8.56 | 7.29 |
| 4 | Hexatriene | 5.16 | 5.09 | 8.23 | 4.91 | 5.54 | 4.81 |
| 5 | Hexatriene | 7.28 | 7.27 | 9.93 | 7.19 | 7.81 | 6.30 |
| 6 | Octatetraene | 6.53 | 6.38 | 9.44 | 6.32 | 6.98 | 5.50 |
| 7 | Octatetraene | 4.56 | 4.44 | 7.86 | 4.22 | 4.88 | 4.12 |
| 8 | Octatetraene | 7.64 | 7.61 | 10.27 | 7.49 | 8.12 | 6.55 |

Table 3: Excitation energies [eV] of 117 singlet excited states on different levels of theory. SOPPA results are taken from S. P. A. Sauer et al., Mol. Phys. 113, 2026 (2015).

| Nr. | molecule | RPA | RPA(D) | HRPA | HRPA(D) | s-HRPA(D) | SOPPA |
|---|---|---|---|---|---|---|---|
| 9 | Octatetraene | 7.46 | 5.57 | 10.68 | 4.91 | 5.70 | 6.14 |
| 10 | Octatetraene | 7.48 | 7.21 | 9.97 | 7.10 | 7.67 | 6.68 |
| 11 | Octatetraene | 8.52 | 6.75 | 11.40 | 6.44 | 7.18 | 7.44 |
| 12 | Cyclopropene | 7.27 | 6.62 | 9.70 | 6.45 | 6.90 | 6.57 |
| 13 | Cyclopropene | 6.60 | 6.88 | 9.07 | 6.69 | 7.18 | 6.65 |
| 14 | Cyclopentadiene | 5.21 | 5.36 | 8.17 | 5.17 | 5.73 | 5.11 |
| 15 | Cyclopentadiene | 8.37 | 6.70 | 11.17 | 6.35 | 7.02 | 6.63 |
| 16 | Cyclopentadiene | 8.39 | 8.46 | 11.00 | 8.31 | 8.96 | 8.32 |
| 17 | Norbornadiene | 5.41 | 5.39 | 8.40 | 5.20 | 5.77 | 5.05 |
| 18 | Norbornadiene | 6.98 | 6.51 | 9.79 | 6.43 | 7.03 | 5.96 |
| 19 | Norbornadiene | 7.80 | 6.98 | 10.67 | 6.67 | 7.30 | 7.12 |
| 20 | Norbornadiene | 8.15 | 7.35 | 10.88 | 7.29 | 7.85 | 7.23 |
| 21 | Benzene | 5.91 | 4.80 | 9.23 | 4.42 | 5.14 | 4.70 |
| 22 | Benzene | 5.93 | 6.50 | 9.15 | 6.22 | 6.92 | 6.15 |
| 23 | Benzene | 7.55 | 7.03 | 10.84 | 6.80 | 7.60 | 6.96 |
| 24 | Benzene | 10.39 | 9.60 | 12.98 | 9.37 | 10.09 | 8.61 |
| 25 | Naphthalene | 5.02 | 3.97 | 8.83 | 3.48 | 4.29 | 3.86 |
| 26 | Naphthalene | 4.76 | 5.02 | 8.47 | 4.68 | 5.46 | 4.41 |
| 27 | Naphthalene | 7.22 | 5.81 | 10.53 | 5.38 | 6.19 | 5.68 |
| 28 | Naphthalene | 6.50 | 6.41 | 9.68 | 6.16 | 6.90 | 5.78 |
| 29 | Naphthalene | 6.60 | 5.86 | 10.37 | 5.58 | 6.50 | 5.74 |
| 30 | Naphthalene | 6.78 | 6.11 | 10.57 | 5.64 | 6.58 | 6.08 |
| 31 | Naphthalene | 7.89 | 5.93 | 11.26 | 5.38 | 6.26 | 6.27 |
| 32 | Naphthalene | 9.16 | 7.96 | 12.25 | 7.29 | 8.17 | 6.91 |
| 33 | Naphthalene | 10.11 | 9.26 | 12.82 | 8.92 | 9.69 | 8.41 |
| 34 | Naphthalene | 9.47 | 7.98 | 12.45 | 7.55 | 8.34 | 8.04 |
| 35 | Furan | 6.17 | 6.46 | 9.42 | 6.32 | 6.99 | 6.23 |
| 36 | Furan | 8.03 | 6.46 | 11.05 | 6.75 | 7.42 | 6.33 |
| 37 | Furan | 8.61 | 8.42 | 11.63 | 7.59 | 8.33 | 8.22 |
| 38 | Pyrrole | 7.48 | 6.28 | 10.57 | 6.06 | 6.73 | 6.09 |
| 39 | Pyrrole | 6.43 | 6.62 | 9.58 | 6.46 | 7.12 | 6.38 |
| 40 | Pyrrole | 8.38 | 8.09 | 11.33 | 7.79 | 8.49 | 7.96 |
| 41 | Imidazole | 7.76 | 6.80 | 10.96 | 6.62 | 7.34 | 6.19 |
| 42 | Imidazole | 7.72 | 6.87 | 10.80 | 6.62 | 7.20 | 6.33 |
| 43 | Imidazole | 6.72 | 6.78 | 9.97 | 6.64 | 7.33 | 6.73 |
| 44 | Imidazole | 8.71 | 7.76 | 11.15 | 7.64 | 8.11 | 7.54 |
| 45 | Imidazole | 8.85 | 8.21 | 12.16 | 7.70 | 8.48 | 8.12 |
| 46 | Pyridine | 5.94 | 4.86 | 9.48 | 4.46 | 5.23 | 4.70 |

Table 3: Excitation energies [eV] of 117 singlet excited states on different levels of theory. SOPPA results are taken from S. P. A. Sauer et al., Mol. Phys. 113, 2026 (2015).

| Nr. | molecule | RPA | RPA(D) | HRPA | HRPA(D) | s-HRPA(D) | SOPPA |
|---|---|---|---|---|---|---|---|
| 47 | Pyridine | 5.95 | 4.94 | 9.11 | 4.57 | 5.12 | 4.59 |
| 48 | Pyridine | 7.38 | 5.13 | 10.27 | 4.64 | 5.19 | 4.91 |
| 49 | Pyridine | 6.17 | 6.72 | 9.51 | 6.40 | 7.13 | 6.31 |
| 50 | Pyridine | 7.83 | 7.30 | 11.28 | 7.06 | 7.92 | 7.20 |
| 51 | Pyridine | 7.94 | 7.14 | 11.35 | 6.89 | 7.73 | 7.10 |
| 52 | Pyridine | 12.12 | 9.79 | 13.56 | 9.82 | 10.53 | 8.93 |
| 53 | Pyridine | 10.55 | 9.71 | 13.41 | 8.95 | 9.59 | 8.76 |
| 54 | Pyrazine | 4.94 | 4.06 | 8.33 | 3.73 | 4.29 | 3.72 |
| 55 | Pyrazine | 6.95 | 4.69 | 9.88 | 4.20 | 4.73 | 4.51 |
| 56 | Pyrazine | 5.66 | 4.69 | 9.53 | 4.25 | 5.10 | 4.48 |
| 57 | Pyrazine | 6.49 | 5.81 | 9.66 | 5.55 | 6.14 | 5.35 |
| 58 | Pyrazine | 9.77 | 6.74 | 12.51 | 5.46 | 6.09 | 6.26 |
| 59 | Pyrazine | 6.28 | 7.00 | 9.76 | 6.62 | 7.39 | 6.53 |
| 60 | Pyrazine | 8.64 | 7.52 | 12.26 | 7.13 | 8.06 | 7.54 |
| 61 | Pyrazine | 8.12 | 7.67 | 11.97 | 7.40 | 8.40 | 7.54 |
| 62 | Pyrazine | 11.12 | 10.39 | 13.97 | 9.93 | 10.65 | 8.94 |
| 63 | Pyrazine | 10.58 | 9.57 | 13.77 | 9.40 | 10.32 | 8.75 |
| 64 | Pyrimidine | 5.71 | 4.30 | 8.92 | 3.90 | 4.44 | 3.94 |
| 65 | Pyrimidine | 6.42 | 4.80 | 9.50 | 4.33 | 4.86 | 4.33 |
| 66 | Pyrimidine | 6.25 | 5.02 | 9.89 | 4.61 | 5.40 | 4.84 |
| 67 | Pyrimidine | 6.54 | 6.94 | 10.00 | 6.58 | 7.34 | 6.50 |
| 68 | Pyrimidine | 8.09 | 7.29 | 11.75 | 6.99 | 7.89 | 7.18 |
| 69 | Pyrimidine | 8.36 | 7.47 | 11.91 | 7.10 | 7.98 | 7.42 |
| 70 | Pyridazine | 4.71 | 3.77 | 8.18 | 3.38 | 3.94 | 3.31 |
| 71 | Pyridazine | 5.91 | 4.74 | 9.08 | 4.15 | 4.70 | 3.92 |
| 72 | Pyridazine | 6.05 | 4.89 | 9.93 | 4.36 | 5.24 | 4.68 |
| 73 | Pyridazine | 7.14 | 5.19 | 10.52 | 4.65 | 5.34 | 5.26 |
| 74 | Pyridazine | 8.36 | 6.09 | 11.29 | 5.62 | 6.24 | 5.92 |
| 75 | Pyridazine | 6.17 | 6.96 | 9.84 | 6.59 | 7.41 | 6.38 |
| 76 | Pyridazine | 7.77 | 7.12 | 11.46 | 6.91 | 7.82 | 7.01 |
| 77 | Pyridazine | 8.11 | 7.41 | 11.72 | 7.16 | 8.08 | 7.35 |
| 78 | s-Triazine | 6.32 | 4.56 | 9.45 | 4.15 | 4.68 | 4.13 |
| 79 | s-Triazine | 6.32 | 4.56 | 9.45 | 4.15 | 4.68 | 4.25 |
| 80 | s-Triazine | 6.13 | 4.55 | 9.29 | 4.14 | 4.67 | 4.21 |
| 81 | s-Triazine | 6.89 | 5.18 | 10.61 | 4.63 | 5.45 | 5.09 |
| 82 | s-Triazine | 7.09 | 7.25 | 10.72 | 6.83 | 7.64 | 6.79 |
| 83 | s-Triazine | 9.57 | 8.03 | 12.32 | 7.67 | 8.27 | 7.49 |
| 84 | s-Triazine | 8.48 | 7.52 | 12.15 | 7.17 | 8.05 | 7.39 |

Table 3: Excitation energies [eV] of 117 singlet excited states on different levels of theory. SOPPA results are taken from S. P. A. Sauer et al., Mol. Phys. 113, 2026 (2015).

| Nr. | molecule | RPA | RPA(D) | HRPA | HRPA(D) | s-HRPA(D) | SOPPA |
|---|---|---|---|---|---|---|---|
| 85 | s-Tetrazine | 3.30 | 2.24 | 7.19 | 1.83 | 2.40 | 1.81 |
| 86 | s-Tetrazine | 5.51 | 3.90 | 8.79 | 3.29 | 3.82 | 3.20 |
| 87 | s-Tetrazine | 5.88 | 5.13 | 9.26 | 4.94 | 5.56 | 4.43 |
| 88 | s-Tetrazine | 5.87 | 4.65 | 10.42 | 3.92 | 5.01 | 4.37 |
| 89 | s-Tetrazine | 6.32 | 5.52 | 9.86 | 5.18 | 5.87 | 4.89 |
| 90 | s-Tetrazine | 6.43 | 4.98 | 10.32 | 4.47 | 5.26 | 4.89 |
| 91 | s-Tetrazine | 9.30 | 5.87 | 12.33 | 5.01 | 5.71 | 5.85 |
| 92 | s-Tetrazine | 9.74 | 5.90 | 12.82 | 4.96 | 5.69 | 6.42 |
| 93 | s-Tetrazine | 12.12 | 7.71 | 15.21 | 6.21 | 6.97 | 7.25 |
| 94 | s-Tetrazine | 8.61 | 6.41 | 11.73 | 5.93 | 6.60 | 6.22 |
| 95 | s-Tetrazine | 6.39 | 7.57 | 12.36 | 6.80 | 7.98 | 6.84 |
| 96 | s-Tetrazine | 8.04 | 7.25 | 10.54 | 7.09 | 8.05 | 7.15 |
| 97 | s-Tetrazine | 9.03 | 7.98 | 13.06 | 7.56 | 8.66 | 7.98 |
| 98 | s-Tetrazine | 10.50 | 9.38 | 14.20 | 9.25 | 10.37 | 8.35 |
| 99 | Formaldehyde | 4.28 | 3.66 | 7.11 | 3.37 | 3.69 | 3.46 |
| 100 | Formaldehyde | 9.36 | 9.01 | 12.43 | 8.65 | 9.23 | 8.71 |
| 101 | Formaldehyde | 12.18 | 9.58 | 13.91 | 9.55 | 9.85 | 9.56 |
| 102 | Acetone | 4.93 | 4.04 | 8.03 | 3.66 | 4.05 | 3.82 |
| 103 | Acetone | 9.54 | 8.95 | 12.73 | 8.56 | 9.18 | 8.66 |
| 104 | Acetone | 9.26 | 8.98 | 12.45 | 8.47 | 9.16 | 8.97 |
| 105 | p-Benzoquinone | 3.78 | 2.56 | 7.32 | 2.10 | 2.58 | 2.10 |
| 106 | p-Benzoquinone | 3.92 | 2.68 | 7.49 | 2.21 | 2.69 | 2.17 |
| 107 | p-Benzoquinone | 4.93 | 4.53 | 8.55 | 4.38 | 5.11 | 4.21 |
| 108 | p-Benzoquinone | 5.88 | 5.21 | 9.90 | 5.10 | 5.99 | 4.76 |
| 109 | p-Benzoquinone | 8.25 | 6.77 | 11.04 | 6.08 | 6.62 | 5.23 |
| 110 | p-Benzoquinone | 8.47 | 6.92 | 11.66 | 6.46 | 7.26 | 6.75 |
| 111 | p-Benzoquinone | 8.36 | 7.98 | 11.17 | 7.32 | 8.03 | 7.75 |
| 112 | Formamide | 6.25 | 5.23 | 9.30 | 4.85 | 5.29 | 5.01 |
| 113 | Formamide | 10.49 | 7.53 | 12.53 | 7.50 | 7.84 | 7.48 |
| 114 | Acetamide | 6.42 | 5.25 | 9.57 | 4.81 | 5.27 | 5.02 |
| 115 | Acetamide | 8.72 | 7.24 | 11.94 | 6.77 | 7.24 | 7.03 |
| 116 | Propanamide | 6.46 | 5.26 | 9.61 | 4.82 | 5.28 | 5.03 |
| 117 | Propanamide | 8.72 | 7.18 | 11.91 | 6.76 | 7.23 | 6.96 |

## 1.4 117 singlet excited states: excitation weights

Table 4: Amount (%) of single excitation character in the excited states at different levels of theory.

| Nr. | molecule | RPA | RPA(D) | HRPA | HRPA(D) | s-HRPA(D) | SOPPA |
|---|---|---|---|---|---|---|---|
| 1 | Ethene | 100.00 | 97.01 | 100.00 | 96.72 | 96.72 | 96.93 |
| 2 | E-Butadiene | 100.00 | 94.97 | 100.00 | 94.48 | 94.48 | 94.08 |
| 3 | E-Butadiene | 100.00 | 95.93 | 100.00 | 95.38 | 95.38 | 89.31 |
| 4 | Hexatriene | 100.00 | 93.75 | 100.00 | 93.03 | 93.03 | 92.68 |
| 5 | Hexatriene | 100.00 | 94.98 | 100.00 | 94.63 | 94.63 | 87.67 |
| 6 | Octatetraene | 100.00 | 93.94 | 100.00 | 93.54 | 93.54 | 86.84 |
| 7 | Octatetraene | 100.00 | 92.94 | 100.00 | 92.01 | 92.01 | 91.71 |
| 8 | Octatetraene | 100.00 | 94.81 | 100.00 | 94.21 | 94.21 | 87.30 |
| 9 | Octatetraene | 100.00 | 85.92 | 100.00 | 81.33 | 81.33 | 92.64 |
| 10 | Octatetraene | 100.00 | 93.49 | 100.00 | 92.84 | 92.84 | 89.41 |
| 11 | Octatetraene | 100.00 | 87.14 | 100.00 | 84.62 | 84.62 | 93.46 |
| 12 | Cyclopropene | 100.00 | 94.29 | 100.00 | 93.69 | 93.69 | 94.34 |
| 13 | Cyclopropene | 100.00 | 96.27 | 100.00 | 95.80 | 95.80 | 95.64 |
| 14 | Cyclopentadiene | 100.00 | 94.88 | 100.00 | 94.31 | 94.31 | 94.11 |
| 15 | Cyclopentadiene | 100.00 | 89.32 | 100.00 | 87.43 | 87.43 | 89.66 |
| 16 | Cyclopentadiene | 100.00 | 95.51 | 100.00 | 94.99 | 94.99 | 95.04 |
| 17 | Norbornadiene | 100.00 | 94.41 | 100.00 | 93.84 | 93.84 | 93.04 |
| 18 | Norbornadiene | 100.00 | 93.82 | 100.00 | 93.49 | 93.49 | 91.44 |
| 19 | Norbornadiene | 100.00 | 92.25 | 100.00 | 90.88 | 90.88 | 93.34 |
| 20 | Norbornadiene | 100.00 | 92.65 | 100.00 | 91.77 | 91.77 | 92.64 |
| 21 | Benzene | 100.00 | 90.08 | 100.00 | 88.15 | 88.15 | 90.12 |
| 22 | Benzene | 100.00 | 94.89 | 100.00 | 94.20 | 94.20 | 93.76 |
| 23 | Benzene | 100.00 | 92.09 | 100.00 | 90.65 | 90.65 | 91.94 |
| 24 | Benzene | 100.00 | 91.55 | 100.00 | 89.90 | 89.90 | 85.04 |
| 25 | Naphthalene | 100.00 | 89.01 | 100.00 | 86.42 | 86.42 | 89.03 |
| 26 | Naphthalene | 100.00 | 92.67 | 100.00 | 91.52 | 91.52 | 90.25 |
| 27 | Naphthalene | 100.00 | 88.64 | 100.00 | 86.23 | 86.23 | 88.70 |
| 28 | Naphthalene | 100.00 | 93.17 | 100.00 | 92.02 | 92.02 | 87.30 |
| 29 | Naphthalene | 100.00 | 90.06 | 100.00 | 88.05 | 88.05 | 89.66 |
| 30 | Naphthalene | 100.00 | 90.06 | 100.00 | 87.37 | 87.37 | 90.42 |
| 31 | Naphthalene | 100.00 | 85.93 | 100.00 | 82.50 | 82.50 | 91.81 |
| 32 | Naphthalene | 100.00 | 88.54 | 100.00 | 84.52 | 84.52 | 83.75 |
| 33 | Naphthalene | 100.00 | 91.28 | 100.00 | 89.18 | 89.18 | 84.84 |
| 34 | Naphthalene | 100.00 | 88.51 | 100.00 | 85.79 | 85.79 | 90.02 |
| 35 | Furan | 100.00 | 94.78 | 100.00 | 94.23 | 94.23 | 93.93 |
| 36 | Furan | 100.00 | 89.39 | 100.00 | 89.47 | 89.47 | 89.55 |
| 37 | Furan | 100.00 | 94.38 | 100.00 | 91.32 | 91.32 | 93.33 |

Table 4: Amount (%) of single excitation character in the excited states at different levels of theory.

| Nr. | molecule | RPA | RPA(D) | HRPA | HRPA(D) | s-HRPA(D) | SOPPA |
|---|---|---|---|---|---|---|---|
| 38 | Pyrrole | 100.00 | 90.26 | 100.00 | 88.85 | 88.85 | 89.89 |
| 39 | Pyrrole | 100.00 | 94.53 | 100.00 | 93.91 | 93.91 | 93.65 |
| 40 | Pyrrole | 100.00 | 93.82 | 100.00 | 92.48 | 92.48 | 93.24 |
| 41 | Imidazole | 100.00 | 91.13 | 100.00 | 89.96 | 89.96 | 90.41 |
| 42 | Imidazole | 100.00 | 92.78 | 100.00 | 91.78 | 91.78 | 90.24 |
| 43 | Imidazole | 100.00 | 94.02 | 100.00 | 93.45 | 93.45 | 92.41 |
| 44 | Imidazole | 100.00 | 92.44 | 100.00 | 91.41 | 91.41 | 91.74 |
| 45 | Imidazole | 100.00 | 92.39 | 100.00 | 90.33 | 90.33 | 92.01 |
| 46 | Pyridine | 100.00 | 90.04 | 100.00 | 88.03 | 88.03 | 89.86 |
| 47 | Pyridine | 100.00 | 91.92 | 100.00 | 90.58 | 90.58 | 90.59 |
| 48 | Pyridine | 100.00 | 89.22 | 100.00 | 87.45 | 87.45 | 89.13 |
| 49 | Pyridine | 100.00 | 94.56 | 100.00 | 93.78 | 93.78 | 93.25 |
| 50 | Pyridine | 100.00 | 91.91 | 100.00 | 90.44 | 90.44 | 91.62 |
| 51 | Pyridine | 100.00 | 91.14 | 100.00 | 89.58 | 89.58 | 91.15 |
| 52 | Pyridine | 100.00 | 85.25 | 100.00 | 90.46 | 90.46 | 84.17 |
| 53 | Pyridine | 100.00 | 90.71 | 100.00 | 87.64 | 87.64 | 85.23 |
| 54 | Pyrazine | 100.00 | 92.09 | 100.00 | 90.95 | 90.95 | 90.90 |
| 55 | Pyrazine | 100.00 | 89.31 | 100.00 | 87.62 | 87.62 | 89.34 |
| 56 | Pyrazine | 100.00 | 89.99 | 100.00 | 87.83 | 87.83 | 89.60 |
| 57 | Pyrazine | 100.00 | 92.72 | 100.00 | 91.72 | 91.72 | 90.30 |
| 58 | Pyrazine | 100.00 | 86.71 | 100.00 | 82.70 | 82.70 | 87.27 |
| 59 | Pyrazine | 100.00 | 94.78 | 100.00 | 94.00 | 94.00 | 93.47 |
| 60 | Pyrazine | 100.00 | 90.05 | 100.00 | 88.05 | 88.05 | 90.53 |
| 61 | Pyrazine | 100.00 | 91.58 | 100.00 | 89.95 | 89.95 | 91.09 |
| 62 | Pyrazine | 100.00 | 92.10 | 100.00 | 89.60 | 89.60 | 83.25 |
| 63 | Pyrazine | 100.00 | 90.12 | 100.00 | 87.41 | 87.41 | 84.50 |
| 64 | Pyrimidine | 100.00 | 90.97 | 100.00 | 89.57 | 89.57 | 89.92 |
| 65 | Pyrimidine | 100.00 | 90.78 | 100.00 | 89.17 | 89.17 | 89.68 |
| 66 | Pyrimidine | 100.00 | 89.76 | 100.00 | 87.76 | 87.76 | 89.55 |
| 67 | Pyrimidine | 100.00 | 93.93 | 100.00 | 92.94 | 92.94 | 92.59 |
| 68 | Pyrimidine | 100.00 | 90.93 | 100.00 | 89.18 | 89.18 | 90.60 |
| 69 | Pyrimidine | 100.00 | 90.97 | 100.00 | 89.18 | 89.18 | 91.06 |
| 70 | Pyridazine | 100.00 | 91.92 | 100.00 | 90.54 | 90.54 | 90.18 |
| 71 | Pyridazine | 100.00 | 91.40 | 100.00 | 89.46 | 89.46 | 88.82 |
| 72 | Pyridazine | 100.00 | 89.27 | 100.00 | 86.86 | 86.86 | 89.05 |
| 73 | Pyridazine | 100.00 | 88.94 | 100.00 | 86.97 | 86.97 | 90.04 |
| 74 | Pyridazine | 100.00 | 89.00 | 100.00 | 87.15 | 87.15 | 88.99 |
| 75 | Pyridazine | 100.00 | 94.44 | 100.00 | 93.69 | 93.69 | 92.25 |
| 76 | Pyridazine | 100.00 | 91.25 | 100.00 | 89.72 | 89.72 | 90.28 |

Table 4: Amount (%) of single excitation character in the excited states at different levels of theory.

| Nr. | molecule | RPA | RPA(D) | HRPA | HRPA(D) | s-HRPA(D) | SOPPA |
|---|---|---|---|---|---|---|---|
| 77 | Pyridazine | 100.00 | 91.21 | 100.00 | 89.67 | 89.67 | 91.18 |
| 78 | s-Triazine | 100.00 | 90.63 | 100.00 | 89.22 | 89.22 | 89.53 |
| 79 | s-Triazine | 100.00 | 90.63 | 100.00 | 89.22 | 89.22 | 89.93 |
| 80 | s-Triazine | 100.00 | 90.72 | 100.00 | 89.29 | 89.29 | 89.72 |
| 81 | s-Triazine | 100.00 | 88.77 | 100.00 | 86.51 | 86.51 | 89.10 |
| 82 | s-Triazine | 100.00 | 93.00 | 100.00 | 91.68 | 91.68 | 91.68 |
| 83 | s-Triazine | 100.00 | 91.66 | 100.00 | 90.28 | 90.28 | 88.65 |
| 84 | s-Triazine | 100.00 | 90.82 | 100.00 | 89.08 | 89.08 | 90.50 |
| 85 | s-Tetrazine | 100.00 | 91.13 | 100.00 | 89.75 | 89.75 | 89.78 |
| 86 | s-Tetrazine | 100.00 | 90.53 | 100.00 | 88.61 | 88.61 | 88.80 |
| 87 | s-Tetrazine | 100.00 | 92.66 | 100.00 | 91.92 | 91.92 | 88.47 |
| 88 | s-Tetrazine | 100.00 | 88.39 | 100.00 | 85.34 | 85.34 | 88.07 |
| 89 | s-Tetrazine | 100.00 | 91.49 | 100.00 | 90.11 | 90.11 | 88.79 |
| 90 | s-Tetrazine | 100.00 | 89.64 | 100.00 | 87.87 | 87.87 | 89.59 |
| 91 | s-Tetrazine | 100.00 | 85.52 | 100.00 | 82.37 | 82.37 | 87.42 |
| 92 | s-Tetrazine | 100.00 | 84.30 | 100.00 | 80.69 | 80.69 | 87.98 |
| 93 | s-Tetrazine | 100.00 | 81.30 | 100.00 | 74.92 | 74.92 | 85.51 |
| 94 | s-Tetrazine | 100.00 | 89.31 | 100.00 | 87.52 | 87.52 | 89.11 |
| 95 | s-Tetrazine | 100.00 | 94.36 | 100.00 | 87.72 | 87.72 | 91.86 |
| 96 | s-Tetrazine | 100.00 | 90.22 | 100.00 | 93.73 | 93.73 | 90.42 |
| 97 | s-Tetrazine | 100.00 | 89.79 | 100.00 | 87.75 | 87.75 | 90.30 |
| 98 | s-Tetrazine | 100.00 | 89.26 | 100.00 | 86.35 | 86.35 | 82.36 |
| 99 | Formaldehyde | 100.00 | 94.38 | 100.00 | 93.73 | 93.73 | 94.15 |
| 100 | Formaldehyde | 100.00 | 94.41 | 100.00 | 93.57 | 93.57 | 93.84 |
| 101 | Formaldehyde | 100.00 | 90.12 | 100.00 | 89.66 | 89.66 | 91.96 |
| 102 | Acetone | 100.00 | 93.52 | 100.00 | 92.59 | 92.59 | 93.28 |
| 103 | Acetone | 100.00 | 93.82 | 100.00 | 92.86 | 92.86 | 93.30 |
| 104 | Acetone | 100.00 | 93.69 | 100.00 | 92.30 | 92.30 | 91.09 |
| 105 | p-Benzoquinone | 100.00 | 91.20 | 100.00 | 89.75 | 89.75 | 89.34 |
| 106 | p-Benzoquinone | 100.00 | 91.05 | 100.00 | 89.61 | 89.61 | 89.00 |
| 107 | p-Benzoquinone | 100.00 | 92.07 | 100.00 | 91.27 | 91.27 | 90.83 |
| 108 | p-Benzoquinone | 100.00 | 90.34 | 100.00 | 89.65 | 89.65 | 89.07 |
| 109 | p-Benzoquinone | 100.00 | 91.09 | 100.00 | 89.05 | 89.05 | 85.67 |
| 110 | p-Benzoquinone | 100.00 | 89.40 | 100.00 | 87.30 | 87.30 | 89.29 |
| 111 | p-Benzoquinone | 100.00 | 93.89 | 100.00 | 91.47 | 91.47 | 91.30 |
| 112 | Formamide | 100.00 | 93.22 | 100.00 | 92.27 | 92.27 | 93.05 |
| 113 | Formamide | 100.00 | 88.53 | 100.00 | 88.14 | 88.14 | 89.21 |
| 114 | Acetamide | 100.00 | 92.85 | 100.00 | 91.76 | 91.76 | 92.67 |
| 115 | Acetamide | 100.00 | 91.26 | 100.00 | 88.85 | 88.85 | 90.88 |

Table 4: Amount (%) of single excitation character in the excited states at different levels of theory.

| Nr. | molecule | RPA | RPA(D) | HRPA | HRPA(D) | s-HRPA(D) | SOPPA |
|---|---|---|---|---|---|---|---|
| 116 | Propanamide | 100.00 | 92.78 | 100.00 | 91.67 | 91.67 | 92.57 |
| 117 | Propanamide | 100.00 | 91.14 | 100.00 | 88.95 | 88.95 | 90.72 |

## 1.5 71 triplet excited states: state info

Table 5: Excited state characteristics of 71 triplet excited states. The light grey colour indicates that the state has an imaginary RPA excitation energy. The dark grey color indicates that the error of the RPA excitation energy is below -3.0 eV.

| Nr. | Molecule | Point group | State | | |
|---|---|---|---|---|---|
| 1 | Ethene | $D_{2h}$ | 1 | $^3B_{1u}$ | $(\pi \rightarrow \pi^*)$ |
| 2 | E-Butadiene | $C_{2h}$ | 1 | $^3B_u$ | $(\pi \rightarrow \pi^*)$ |
| 3 | E-Butadiene | $C_{2h}$ | 1 | $^3A_g$ | $(\pi \rightarrow \pi^*)$ |
| 4 | Hexatriene | $C_{2h}$ | 1 | $^3B_u$ | $(\pi \rightarrow \pi^*)$ |
| 5 | Hexatriene | $C_{2h}$ | 1 | $^3A_g$ | $(\pi \rightarrow \pi^*)$ |
| 6 | Octatetraene | $C_{2h}$ | 1 | $^3B_u$ | $(\pi \rightarrow \pi^*)$ |
| 7 | Octatetraene | $C_{2h}$ | 1 | $^3A_g$ | $(\pi \rightarrow \pi^*)$ |
| 8 | Cyclopropene | $C_{2v}$ | 1 | $^3B_2$ | $(\pi \rightarrow \pi^*)$ |
| 9 | Cyclopropene | $C_{2v}$ | 1 | $^3B_1$ | $(\sigma \rightarrow \pi^*)$ |
| 10 | Cyclopentadiene | $C_{2v}$ | 1 | $^3B_2$ | $(\pi \rightarrow \pi^*)$ |
| 11 | Cyclopentadiene | $C_{2v}$ | 1 | $^3A_1$ | $(\pi \rightarrow \pi^*)$ |
| 12 | Norbornadiene | $C_{2v}$ | 1 | $^3A_2$ | $(\pi \rightarrow \pi^*)$ |
| 13 | Norbornadiene | $C_{2v}$ | 1 | $^3B_2$ | $(\pi \rightarrow \pi^*)$ |
| 14 | Benzene | $D_{2h}$ | 1 | $^3B_{2u}$ | $(\pi \rightarrow \pi^*)$ |
| 15 | Benzene | $D_{2h}$ | 1 | $^3B_{3u}$ | $(\pi \rightarrow \pi^*)$ |
| 16 | Benzene | $D_{2h}$ | 2 | $^3B_{3u}$ | $(\pi \rightarrow \pi^*)$ |
| 17 | Benzene | $D_{2h}$ | 1 | $^3A_g$ | $(\pi \rightarrow \pi^*)$ |
| 18 | Naphthalene | $D_{2h}$ | 1 | $^3B_{2u}$ | $(\pi \rightarrow \pi^*)$ |
| 19 | Naphthalene | $D_{2h}$ | 1 | $^3B_{3u}$ | $(\pi \rightarrow \pi^*)$ |
| 20 | Naphthalene | $D_{2h}$ | 1 | $^3B_{1g}$ | $(\pi \rightarrow \pi^*)$ |
| 21 | Naphthalene | $D_{2h}$ | 2 | $^3B_{2u}$ | $(\pi \rightarrow \pi^*)$ |
| 22 | Naphthalene | $D_{2h}$ | 2 | $^3B_{3u}$ | $(\pi \rightarrow \pi^*)$ |
| 23 | Naphthalene | $D_{2h}$ | 1 | $^3A_g$ | $(\pi \rightarrow \pi^*)$ |
| 24 | Naphthalene | $D_{2h}$ | 2[a] | $^3B_{1g}$ | $(\pi \rightarrow \pi^*)$ |
| 25 | Naphthalene | $D_{2h}$ | 2 | $^3A_g$ | $(\pi \rightarrow \pi^*)$ |
| 26 | Naphthalene | $D_{2h}$ | 3 | $^3A_g$ | $(\pi \rightarrow \pi^*)$ |
| 27 | Naphthalene | $D_{2h}$ | 3[b] | $^3B_{1g}$ | $(\pi \rightarrow \pi^*)$ |
| 28 | Furan | $C_{2v}$ | 1 | $^3B_2$ | $(\pi \rightarrow \pi^*)$ |

Table 5: Excited state characteristics of 71 triplet excited states. The light grey colour indicates that the state has an imaginary RPA excitation energy. The dark grey color indicates that the error of the RPA excitation energy is below -3.0 eV.

| Nr. | Molecule | Point group | State | | |
|---|---|---|---|---|---|
| 29 | Furan | $C_{2v}$ | 1 | $^3A_1$ | $(\pi \rightarrow \pi^*)$ |
| 30 | Pyrrole | $C_{2v}$ | 1 | $^3B_2$ | $(\pi \rightarrow \pi^*)$ |
| 31 | Pyrrole | $C_{2v}$ | 1 | $^3A_1$ | $(\pi \rightarrow \pi^*)$ |
| 32 | Imidazole | $C_s$ | 1 | $^3A'$ | $(\pi \rightarrow \pi^*)$ |
| 33 | Imidazole | $C_s$ | 2 | $^3A'$ | $(\pi \rightarrow \pi^*)$ |
| 34 | Imidazole | $C_s$ | $1^c$ | $^3A$” | $(n \rightarrow \pi^*)$ |
| 35 | Imidazole | $C_s$ | 3 | $^3A'$ | $(\pi \rightarrow \pi^*)$ |
| 36 | Imidazole | $C_s$ | 4 | $^3A'$ | $(\pi \rightarrow \pi^*)$ |
| 37 | Imidazole | $C_s$ | $3^d$ | $^3A$” | $(n \rightarrow \pi^*)$ |
| 38 | Pyridine | $C_{2v}$ | 1 | $^3A_1$ | $(\pi \rightarrow \pi^*)$ |
| 39 | Pyridine | $C_{2v}$ | 1 | $^3B_1$ | $(n \rightarrow \pi^*)$ |
| 40 | Pyridine | $C_{2v}$ | 1 | $^3B_2$ | $(\pi \rightarrow \pi^*)$ |
| 41 | Pyridine | $C_{2v}$ | 2 | $^3A_1$ | $(\pi \rightarrow \pi^*)$ |
| 42 | Pyridine | $C_{2v}$ | 1 | $^3A_2$ | $(n \rightarrow \pi^*)$ |
| 43 | Pyridine | $C_{2v}$ | 2 | $^3B_2$ | $(\pi \rightarrow \pi^*)$ |
| 44 | Pyridine | $C_{2v}$ | 3 | $^3B_2$ | $(\pi \rightarrow \pi^*)$ |
| 45 | Pyridine | $C_{2v}$ | 3 | $^3A_1$ | $(\pi \rightarrow \pi^*)$ |
| 46 | s-Tetrazine | $D_{2h}$ | 1 | $^3B_{3u}$ | $(n \rightarrow \pi^*)$ |
| 47 | s-Tetrazine | $D_{2h}$ | 1 | $^3A_u$ | $(n \rightarrow \pi^*)$ |
| 48 | s-Tetrazine | $D_{2h}$ | 1 | $^3B_{1g}$ | $(n \rightarrow \pi^*)$ |
| 49 | s-Tetrazine | $D_{2h}$ | 1 | $^3B_{1u}$ | $(\pi \rightarrow \pi^*)$ |
| 50 | s-Tetrazine | $D_{2h}$ | 1 | $^3B_{2u}$ | $(\pi \rightarrow \pi^*)$ |
| 51 | s-Tetrazine | $D_{2h}$ | 1 | $^3B_{2g}$ | $(n \rightarrow \pi^*)$ |
| 52 | s-Tetrazine | $D_{2h}$ | 2 | $^3A_u$ | $(n \rightarrow \pi^*)$ |
| 53 | s-Tetrazine | $D_{2h}$ | 2 | $^3B_{1u}$ | $(\pi \rightarrow \pi^*)$ |
| 54 | s-Tetrazine | $D_{2h}$ | 2 | $^3B_{2g}$ | $(n \rightarrow \pi^*)$ |
| 55 | s-Tetrazine | $D_{2h}$ | 2 | $^3B_{1g}$ | $(n \rightarrow \pi^*)$ |
| 56 | s-Tetrazine | $D_{2h}$ | 2 | $^3B_{3u}$ | $(n \rightarrow \pi^*)$ |
| 57 | s-Tetrazine | $D_{2h}$ | 2 | $^3B_{2u}$ | $(\pi \rightarrow \pi^*)$ |
| 58 | Formaldehyde | $C_{2v}$ | 1 | $^3A_2$ | $(\pi \rightarrow \pi^*)$ |
| 59 | Formaldehyde | $C_{2v}$ | 1 | $^3A_1$ | $(\pi \rightarrow \pi^*)$ |
| 60 | Acetone | $C_{2v}$ | 1 | $^3A_2$ | $(n \rightarrow \pi^*)$ |
| 61 | Acetone | $C_{2v}$ | 1 | $^3A_1$ | $(\pi \rightarrow \pi^*)$ |
| 62 | p-Benzoquinone | $D_{2h}$ | 1 | $^3B_{1g}$ | $(n \rightarrow \pi^*)$ |
| 63 | p-Benzoquinone | $D_{2h}$ | 1 | $^3A_u$ | $(n \rightarrow \pi^*)$ |
| 64 | p-Benzoquinone | $D_{2h}$ | 1 | $^3B_{1u}$ | $(\pi \rightarrow \pi^*)$ |
| 65 | p-Benzoquinone | $D_{2h}$ | 1 | $^3B_{3g}$ | $(\pi \rightarrow \pi^*)$ |

Table 5: Excited state characteristics of 71 triplet excited states. The light grey colour indicates that the state has an imaginary RPA excitation energy. The dark grey color indicates that the error of the RPA excitation energy is below -3.0 eV.

| Nr. | Molecule | Point group | State | | |
|---|---|---|---|---|---|
| 66 | Formamide | $C_s$ | 1 | $^3A$" | $(n \rightarrow \pi^*)$ |
| 67 | Formamide | $C_s$ | 1 | $^3A'$ | $(\pi \rightarrow \pi^*)$ |
| 68 | Acetamide | $C_s$ | 1 | $^3A$" | $(n \rightarrow \pi^*)$ |
| 69 | Acetamide | $C_s$ | 1 | $^3A'$ | $(\pi \rightarrow \pi^*)$ |
| 70 | Propanamide | $C_s$ | 1 | $^3A$" | $(n \rightarrow \pi^*)$ |
| 71 | Propanamide | $C_s$ | 1 | $^3A'$ | $(\pi \rightarrow \pi^*)$ |

[a] RPA and HRPA state nr. 3

[b] RPA and HRPA state nr. 2

[c] HRPA state nr. 2

[d] RPA and HRPA state nr. 8

## 1.6 71 triplet excited states: Excitation energies

Table 6: Excitation energies [eV] of 71 triplet excited states on different levels of theory. SOPPA results are taken from S. P. A. Sauer et al., Mol. Phys. 113, 2026 (2015).

| Nr. | molecule | RPA | RPA(D) | HRPA | HRPA(D) | s-HRPA(D) | SOPPA |
|---|---|---|---|---|---|---|---|
| 1 | Ethene | -0.00 | 13.28 | 5.47 | 3.95 | 4.25 | 3.95 |
| 2 | E-Butadiene | -0.00 | 3.70 | 4.86 | 2.87 | 3.19 | 2.77 |
| 3 | E-Butadiene | 2.83 | 5.60 | 6.48 | 4.66 | 5.05 | 4.68 |
| 4 | Hexatriene | 0.00 | 2.45 | 4.60 | 2.31 | 2.66 | 2.13 |
| 5 | Hexatriene | 1.31 | 7.18 | 5.86 | 3.86 | 4.24 | 3.80 |
| 6 | Octatetraene | 0.00 | 3.16 | 4.46 | 1.98 | 2.34 | 1.74 |
| 7 | Octatetraene | -0.00 | 17.25 | 5.44 | 3.27 | 3.65 | 3.14 |
| 8 | Cyclopropene | 0.00 | 11.77 | 5.69 | 3.92 | 4.26 | 3.90 |
| 9 | Cyclopropene | 6.65 | 6.30 | 9.11 | 6.15 | 6.58 | 6.24 |
| 10 | Cyclopentadiene | 0.00 | 8.33 | 5.02 | 2.84 | 3.20 | 2.75 |
| 11 | Cyclopentadiene | 2.86 | 5.57 | 6.68 | 4.62 | 5.05 | 4.63 |
| 12 | Norbornadiene | 0.00 | 4.24 | 5.45 | 3.26 | 3.64 | 3.16 |
| 13 | Norbornadiene | -0.00 | 5.52 | 5.73 | 3.68 | 4.08 | 3.64 |
| 14 | Benzene | 0.00 | 11.30 | 5.94 | 3.82 | 4.28 | 3.73 |
| 15 | Benzene | 4.76 | 4.65 | 8.04 | 4.40 | 5.03 | 4.56 |
| 16 | Benzene | 5.30 | 5.95 | 8.80 | 5.60 | 6.28 | 5.66 |
| 17 | Benzene | 7.33 | 7.64 | 10.17 | 7.40 | 8.00 | 7.47 |
| 18 | Naphthalene | -0.00 | 3.17 | 5.50 | 2.96 | 3.45 | 2.68 |
| 19 | Naphthalene | 3.90 | 4.10 | 7.58 | 3.85 | 4.51 | 3.78 |

Table 6: Excitation energies [eV] of 71 triplet excited states on different levels of theory. SOPPA results are taken from S. P. A. Sauer et al., Mol. Phys. 113, 2026 (2015).

| Nr. | molecule | RPA | RPA(D) | HRPA | HRPA(D) | s-HRPA(D) | SOPPA |
|---|---|---|---|---|---|---|---|
| 20 | Naphthalene | 2.43 | 5.37 | 6.83 | 4.11 | 4.65 | 4.05 |
| 21 | Naphthalene | 4.40 | 4.27 | 8.21 | 3.98 | 4.74 | 4.29 |
| 22 | Naphthalene | 4.53 | 5.03 | 8.49 | 4.59 | 5.37 | 4.66 |
| 23 | Naphthalene | 4.85 | 5.43 | 8.19 | 5.14 | 5.75 | 5.15 |
| 24 | Naphthalene | 7.77 | 6.17 | 11.09 | 5.72 | 6.57 | 6.01 |
| 25 | Naphthalene | 6.70 | 6.59 | 10.04 | 6.31 | 7.05 | 6.37 |
| 26 | Naphthalene | 7.05 | 6.69 | 10.42 | 6.32 | 7.11 | 6.46 |
| 27 | Naphthalene | 7.09 | 6.70 | 10.22 | 6.44 | 7.16 | 6.70 |
| 28 | Furan | 0.00 | 4.56 | 6.10 | 3.87 | 4.32 | 3.77 |
| 29 | Furan | 4.07 | 5.45 | 7.59 | 5.09 | 5.59 | 5.03 |
| 30 | Pyrrole | 1.30 | 8.50 | 6.47 | 4.21 | 4.67 | 4.11 |
| 31 | Pyrrole | 4.68 | 5.40 | 7.95 | 5.18 | 5.70 | 5.13 |
| 32 | Imidazole | 1.51 | 8.60 | 6.76 | 4.41 | 4.89 | 4.31 |
| 33 | Imidazole | 4.56 | 5.89 | 8.36 | 5.44 | 6.03 | 5.40 |
| 34 | Imidazole | 6.42 | 6.19 | 9.51 | 5.93 | 6.44 | 5.86 |
| 35 | Imidazole | 5.86 | 6.52 | 9.68 | 6.11 | 6.77 | 6.22 |
| 36 | Imidazole | 7.64 | 7.15 | 11.01 | 6.91 | 7.61 | 7.16 |
| 37 | Imidazole | 9.12 | 7.69 | 12.04 | 7.10 | 7.72 | 7.08 |
| 38 | Pyridine | -0.00 | 4.10 | 6.20 | 3.98 | 4.46 | 3.88 |
| 39 | Pyridine | 4.77 | 4.33 | 7.92 | 4.03 | 4.50 | 3.97 |
| 40 | Pyridine | 4.46 | 4.79 | 8.08 | 4.39 | 5.06 | 4.48 |
| 41 | Pyridine | 4.82 | 4.82 | 8.32 | 4.56 | 5.23 | 4.71 |
| 42 | Pyridine | 7.08 | 5.17 | 9.99 | 4.73 | 5.27 | 4.88 |
| 43 | Pyridine | 5.97 | 6.17 | 9.42 | 6.00 | 6.70 | 6.03 |
| 44 | Pyridine | 7.91 | 7.96 | 10.90 | 7.59 | 8.27 | 7.84 |
| 45 | Pyridine | 7.23 | 7.82 | 10.46 | 7.53 | 8.21 | 7.59 |
| 46 | s-Tetrazine | 1.84 | 1.67 | 5.80 | 1.33 | 1.77 | 1.13 |
| 47 | s-Tetrazine | 4.33 | 3.72 | 7.68 | 3.26 | 3.74 | 2.92 |
| 48 | s-Tetrazine | 4.01 | 3.95 | 7.39 | 3.77 | 4.25 | 3.54 |
| 49 | s-Tetrazine | -0.00 | 3.92 | 6.54 | 4.14 | 4.76 | 3.94 |
| 50 | s-Tetrazine | 3.41 | 4.63 | 8.30 | 3.92 | 4.81 | 4.10 |
| 51 | s-Tetrazine | 4.93 | 4.82 | 8.39 | 4.55 | 5.11 | 4.33 |
| 52 | s-Tetrazine | 5.86 | 4.49 | 9.72 | 3.92 | 4.62 | 4.41 |
| 53 | s-Tetrazine | 5.37 | 5.00 | 9.55 | 4.53 | 5.47 | 4.89 |
| 54 | s-Tetrazine | 8.76 | 6.06 | 11.93 | 5.25 | 5.95 | 5.57 |
| 55 | s-Tetrazine | 9.58 | 5.90 | 12.71 | 5.00 | 5.74 | 6.24 |
| 56 | s-Tetrazine | 8.06 | 6.31 | 11.16 | 5.93 | 6.57 | 6.03 |
| 57 | s-Tetrazine | 7.26 | 7.25 | 11.41 | 6.78 | 7.79 | 7.02 |

Table 6: Excitation energies [eV] of 71 triplet excited states on different levels of theory. SOPPA results are taken from S. P. A. Sauer et al., Mol. Phys. 113, 2026 (2015).

| Nr. | molecule | RPA | RPA(D) | HRPA | HRPA(D) | s-HRPA(D) | SOPPA |
|---|---|---|---|---|---|---|---|
| 58 | Formaldehyde | 3.33 | 3.18 | 6.23 | 2.90 | 3.18 | 2.94 |
| 59 | Formaldehyde | 0.91 | 19.59 | 7.48 | 5.41 | 5.88 | 5.43 |
| 60 | Acetone | 4.08 | 3.65 | 7.24 | 3.29 | 3.65 | 3.39 |
| 61 | Acetone | 2.77 | 8.27 | 8.14 | 5.59 | 6.10 | 5.61 |
| 62 | p-Benzoquinone | 2.95 | 2.25 | 6.54 | 1.82 | 2.25 | 1.75 |
| 63 | p-Benzoquinone | 3.07 | 2.38 | 6.69 | 1.94 | 2.38 | 1.84 |
| 64 | p-Benzoquinone | -0.00 | 3.14 | 5.45 | 2.88 | 3.32 | 2.44 |
| 65 | p-Benzoquinone | 0.00 | 3.20 | 5.66 | 3.13 | 3.58 | 2.89 |
| 66 | Formamide | 5.48 | 4.94 | 8.59 | 4.59 | 4.99 | 4.67 |
| 67 | Formamide | 4.09 | 6.59 | 8.56 | 5.36 | 5.86 | 5.35 |
| 68 | Acetamide | 5.68 | 4.98 | 8.88 | 4.58 | 5.01 | 4.70 |
| 69 | Acetamide | 4.42 | 6.60 | 8.84 | 5.48 | 5.99 | 5.46 |
| 70 | Propanamide | 5.71 | 5.00 | 8.93 | 4.60 | 5.03 | 4.71 |
| 71 | Propanamide | 4.49 | 6.59 | 8.89 | 5.48 | 6.00 | 5.47 |

## 1.7 71 triplet excited states: excitation weights

Table 7: Amount (%) of single excitation character in the excited states at different levels of theory.

| Nr. | molecule | RPA | RPA(D) | HRPA | HRPA(D) | s-HRPA(D) | SOPPA |
|---|---|---|---|---|---|---|---|
| 1 | Ethene | 100.00 | 98.26 | 100.00 | 97.90 | 97.90 | 97.97 |
| 2 | E-Butadiene | 100.00 | 97.46 | 100.00 | 96.93 | 96.93 | 96.61 |
| 3 | E-Butadiene | 100.00 | 97.52 | 100.00 | 97.12 | 97.12 | 97.24 |
| 4 | Hexatriene | 100.00 | 96.90 | 100.00 | 96.25 | 96.25 | 95.64 |
| 5 | Hexatriene | 100.00 | 97.28 | 100.00 | 96.76 | 96.76 | 96.51 |
| 6 | Octatetraene | 100.00 | 96.54 | 100.00 | 95.78 | 95.78 | 94.93 |
| 7 | Octatetraene | 100.00 | 96.98 | 100.00 | 96.37 | 96.37 | 95.83 |
| 8 | Cyclopropene | 100.00 | 97.90 | 100.00 | 97.37 | 97.37 | 97.30 |
| 9 | Cyclopropene | 100.00 | 94.99 | 100.00 | 94.47 | 94.47 | 94.95 |
| 10 | Cyclopentadiene | 100.00 | 97.21 | 100.00 | 96.56 | 96.56 | 96.23 |
| 11 | Cyclopentadiene | 100.00 | 97.12 | 100.00 | 96.61 | 96.61 | 96.71 |
| 12 | Norbornadiene | 100.00 | 97.19 | 100.00 | 96.50 | 96.50 | 96.04 |
| 13 | Norbornadiene | 100.00 | 97.51 | 100.00 | 96.93 | 96.93 | 96.74 |
| 14 | Benzene | 100.00 | 97.27 | 100.00 | 96.67 | 96.67 | 96.28 |
| 15 | Benzene | 100.00 | 93.29 | 100.00 | 92.13 | 92.13 | 93.13 |
| 16 | Benzene | 100.00 | 94.81 | 100.00 | 94.08 | 94.08 | 94.51 |
| 17 | Benzene | 100.00 | 94.78 | 100.00 | 93.77 | 93.77 | 94.32 |

Table 7: Amount (%) of single excitation character in the excited states at different levels of theory.

| Nr. | molecule | RPA | RPA(D) | HRPA | HRPA(D) | s-HRPA(D) | SOPPA |
|---|---|---|---|---|---|---|---|
| 18 | Naphthalene | 100.00 | 96.58 | 100.00 | 95.51 | 95.51 | 93.94 |
| 19 | Naphthalene | 100.00 | 92.77 | 100.00 | 91.63 | 91.63 | 91.66 |
| 20 | Naphthalene | 100.00 | 95.76 | 100.00 | 94.77 | 94.77 | 94.54 |
| 21 | Naphthalene | 100.00 | 91.71 | 100.00 | 90.20 | 90.20 | 92.24 |
| 22 | Naphthalene | 100.00 | 93.22 | 100.00 | 92.01 | 92.01 | 92.41 |
| 23 | Naphthalene | 100.00 | 94.67 | 100.00 | 93.70 | 93.70 | 93.85 |
| 24 | Naphthalene | 100.00 | 87.53 | 100.00 | 84.67 | 84.67 | 87.85 |
| 25 | Naphthalene | 100.00 | 92.53 | 100.00 | 91.02 | 91.02 | 91.40 |
| 26 | Naphthalene | 100.00 | 91.67 | 100.00 | 90.04 | 90.04 | 91.18 |
| 27 | Naphthalene | 100.00 | 92.29 | 100.00 | 90.88 | 90.88 | 92.40 |
| 28 | Furan | 100.00 | 97.31 | 100.00 | 96.67 | 96.67 | 96.27 |
| 29 | Furan | 100.00 | 96.15 | 100.00 | 95.63 | 95.63 | 95.46 |
| 30 | Pyrrole | 100.00 | 97.12 | 100.00 | 96.51 | 96.51 | 96.08 |
| 31 | Pyrrole | 100.00 | 95.40 | 100.00 | 94.78 | 94.78 | 94.78 |
| 32 | Imidazole | 100.00 | 97.10 | 100.00 | 96.40 | 96.40 | 95.93 |
| 33 | Imidazole | 100.00 | 95.72 | 100.00 | 94.86 | 94.86 | 94.76 |
| 34 | Imidazole | 100.00 | 94.32 | 100.00 | 93.57 | 93.57 | 92.83 |
| 35 | Imidazole | 100.00 | 94.48 | 100.00 | 93.63 | 93.63 | 94.30 |
| 36 | Imidazole | 100.00 | 92.62 | 100.00 | 91.66 | 91.66 | 93.17 |
| 37 | Imidazole | 100.00 | 91.18 | 100.00 | 89.28 | 89.28 | 90.73 |
| 38 | Pyridine | 100.00 | 97.20 | 100.00 | 96.52 | 96.52 | 96.09 |
| 39 | Pyridine | 100.00 | 93.42 | 100.00 | 92.49 | 92.49 | 92.12 |
| 40 | Pyridine | 100.00 | 93.85 | 100.00 | 92.59 | 92.59 | 93.16 |
| 41 | Pyridine | 100.00 | 93.30 | 100.00 | 92.14 | 92.14 | 93.07 |
| 42 | Pyridine | 100.00 | 89.99 | 100.00 | 88.40 | 88.40 | 89.58 |
| 43 | Pyridine | 100.00 | 93.82 | 100.00 | 93.29 | 93.29 | 93.89 |
| 44 | Pyridine | 100.00 | 94.03 | 100.00 | 92.62 | 92.62 | 93.87 |
| 45 | Pyridine | 100.00 | 94.89 | 100.00 | 93.80 | 93.80 | 93.68 |
| 46 | s-Tetrazine | 100.00 | 92.93 | 100.00 | 92.04 | 92.04 | 91.29 |
| 47 | s-Tetrazine | 100.00 | 92.85 | 100.00 | 91.53 | 91.53 | 90.10 |
| 48 | s-Tetrazine | 100.00 | 94.42 | 100.00 | 93.88 | 93.88 | 92.17 |
| 49 | s-Tetrazine | 100.00 | 97.13 | 100.00 | 96.66 | 96.66 | 95.57 |
| 50 | s-Tetrazine | 100.00 | 93.46 | 100.00 | 91.95 | 91.95 | 92.87 |
| 51 | s-Tetrazine | 100.00 | 93.51 | 100.00 | 92.66 | 92.66 | 91.48 |
| 52 | s-Tetrazine | 100.00 | 89.81 | 100.00 | 87.98 | 87.98 | 90.47 |
| 53 | s-Tetrazine | 100.00 | 91.64 | 100.00 | 89.72 | 89.72 | 91.70 |
| 54 | s-Tetrazine | 100.00 | 87.05 | 100.00 | 84.22 | 84.22 | 87.36 |
| 55 | s-Tetrazine | 100.00 | 84.52 | 100.00 | 81.06 | 81.06 | 88.13 |
| 56 | s-Tetrazine | 100.00 | 90.42 | 100.00 | 88.98 | 88.98 | 89.88 |

Table 7: Amount (%) of single excitation character in the excited states at different levels of theory.

| Nr. | molecule | RPA | RPA(D) | HRPA | HRPA(D) | s-HRPA(D) | SOPPA |
|---|---|---|---|---|---|---|---|
| 57 | s-Tetrazine | 100.00 | 92.30 | 100.00 | 90.92 | 90.92 | 92.23 |
| 58 | Formaldehyde | 100.00 | 95.24 | 100.00 | 94.74 | 94.74 | 94.94 |
| 59 | Formaldehyde | 100.00 | 97.90 | 100.00 | 97.40 | 97.40 | 97.50 |
| 60 | Acetone | 100.00 | 94.39 | 100.00 | 93.64 | 93.64 | 94.06 |
| 61 | Acetone | 100.00 | 97.15 | 100.00 | 96.54 | 96.54 | 96.59 |
| 62 | p-Benzoquinone | 100.00 | 92.44 | 100.00 | 91.30 | 91.30 | 90.51 |
| 63 | p-Benzoquinone | 100.00 | 92.37 | 100.00 | 91.25 | 91.25 | 90.26 |
| 64 | p-Benzoquinone | 100.00 | 96.07 | 100.00 | 95.99 | 95.99 | 94.15 |
| 65 | p-Benzoquinone | 100.00 | 96.49 | 100.00 | 95.80 | 95.80 | 94.76 |
| 66 | Formamide | 100.00 | 94.13 | 100.00 | 93.38 | 93.38 | 93.80 |
| 67 | Formamide | 100.00 | 96.10 | 100.00 | 95.26 | 95.26 | 95.29 |
| 68 | Acetamide | 100.00 | 93.76 | 100.00 | 92.87 | 92.87 | 93.41 |
| 69 | Acetamide | 100.00 | 95.90 | 100.00 | 95.00 | 95.00 | 94.95 |
| 70 | Propanamide | 100.00 | 93.70 | 100.00 | 92.79 | 92.79 | 93.32 |
| 71 | Propanamide | 100.00 | 95.81 | 100.00 | 94.89 | 94.89 | 94.84 |

## 1.8 Timings

| | | RPA(D) | HRPA(D) | SOPPA |
|---|---|---|---|---|
| Benzene | singlet | 01:21 | 05:55 | 10:21 |
| | triplet | 01:13 | 03:55 | 08:43 |
| Naphthalene | singlet | 08:17 | 41:15 | 72:03 |
| | triplet | 09:33 | 34:28 | 75:52 |

Table 8: Absolute total timings (mm:ss) of the RPA(D), HRPA(D) and SOPPA methods.
